# 2-Aryladenine Derivatives as a Potent Scaffold for Adenosine Receptor Antagonists: The 6-Morpholino Derivatives

**DOI:** 10.3390/molecules29112543

**Published:** 2024-05-28

**Authors:** Filipe Areias, Carla Correia, Ashly Rocha, Sofia Teixeira, Marián Castro, Jose Brea, Huabin Hu, Jens Carlsson, Maria I. Loza, M. Fernanda Proença, M. Alice Carvalho

**Affiliations:** 1 Centre of Chemistry of University of Minho (CQUM), Campus de Gualtar, Universidade do Minho, 4710-057 Braga, Portugal; filipeareias@yahoo.com.br (F.A.); carlabcpereira@gmail.com (C.C.); ashlyrochaa@gmail.com (A.R.); id9191@alunos.uminho.pt (S.T.); fproenca@quimica.uminho.pt (M.F.P.); 2 Center for Research in Molecular Medicine and Chronic Diseases (CiMUS), Universidade de Santiago de Compostela, Avda de Barcelona, E-15782 Santiago de Compostela, Spain; marian.castro@usc.es (M.C.); pepo.brea@usc.es (J.B.); mabel.loza@usc.es (M.I.L.); 3 School of Chemical Sciences & Engineering, Yachay Tech University, Yachay City of Knowledge, Urcuqui 100119, Ecuador; 4 Instituto de Investigación Sanitaria de Santiago de Compostela (IDIS), Travesía da Choupana s/n, E-15706 Santiago de Compostela, Spain; 5 Science for Life Laboratory, Department of Cell and Molecular Biology, Uppsala University, SE-75124 Uppsala, Sweden; huabin.hu@icm.uu.se (H.H.); jens.carlsson.lab@gmail.com (J.C.)

**Keywords:** G protein-coupled receptors, adenine derivatives, adenosine receptor antagonists, 2-arylpurine derivatives, structure-activity relationship

## Abstract

A set of 2-aryl-9-H or methyl-6-morpholinopurine derivatives were synthesized and assayed through radioligand binding tests at human A_1_, A_2A_, A_2B_, and A_3_ adenosine receptor subtypes. Eleven purines showed potent antagonism at A_1_, A_3_, dual A_1_/A_2A_, A_1_/A_2B_, or A_1_/A_3_ adenosine receptors. Additionally, three compounds showed high affinity without selectivity for any specific adenosine receptor. The structure-activity relationships were made for this group of new compounds. The 9-methylpurine derivatives were generally less potent but more selective, and the 9H-purine derivatives were more potent but less selective. These compounds can be an important source of new biochemical tools and/or pharmacological drugs.

## 1. Introduction

Adenosine, a purine nucleoside composed of an adenine linked to a ribose via a β-N9-glycosidic bond, modulates many physiological conditions related to neurological, immunologic, and cardiovascular systems [1,2]. Adenosine receptors, namely A_1_, A_2A_, A_2B_, and A_3_, belong to the G protein-coupled receptor (GPCR) superfamily and are widely recognized as attractive targets for the design and development of new therapeutic agents against different clinical disorders [3,4,5]. The activation of the A_1_ and A_3_ receptors inhibits adenylyl cyclase activity through the action of G_i/o_ proteins and, as a result, intracellular cyclic AMP (cAMP) decreases, while the A_2A_ and A_2B_ receptors increase cAMP production through the action of G_s_ proteins [6]. So, it is of therapeutic importance to synthesize new drugs active on adenosine receptors A_1_, A_3_, and, additionally, dual A_1_/A_3_ to study the synergistic effect between these two receptor subtypes as they preferentially couple to the same G proteins [2]. Indeed, there are many patents for adenosine receptor ligands, and some of these ligands are already in clinical trials or waiting for FDA approval [4,5,7]. For example, A_1_ ligands are useful for glaucoma, heart failure, angina, seizures, ischemia, depression, obesity, asthma, renal protection, edema associated with congestive heart failure, atrial arrhythmias, type II diabetes, neuropathic pain, neuroprotection, atrial fibrillation, tachycardia, cardioprotection, and sleep regulation [1,8,9,10,11,12]. In addition, A_3_ ligands are useful for liver regeneration, hepatitis, psoriasis, rheumatoid arthritis, inflammation, dry eye syndrome, fibrotic diseases, neurodegeneration, ischaemia, asthma, chronic obstructive pulmonary disease, glaucoma, and cancer [13,14,15,16,17,18,19,20], with A_3_ antagonist PBF-677 (Palobiofarma SL), that has reached clinical trials for glaucoma, ulcerative colitis, and eosinophilic esophagitis [21,22]. Finally, dual A_1_/A_3_ ligands can be useful as potential therapeutics for treating glaucoma, kidney failure, pulmonary diseases, and Alzheimer’s disease [23,24]. Despite the fact that several adenosine receptor ligands are already in clinical trials, achieving selectivity is still a challenge. Additionally, a number of side effects were reported for A_1_ antagonists in the literature, such as an increase in the frequency of seizures, strokes [25,26], dizziness, nausea, transient hypertension, and transient hypotension [27] in treated patients, which justifies the search for new high-affinity and selective drugs.

**Figure 1:**
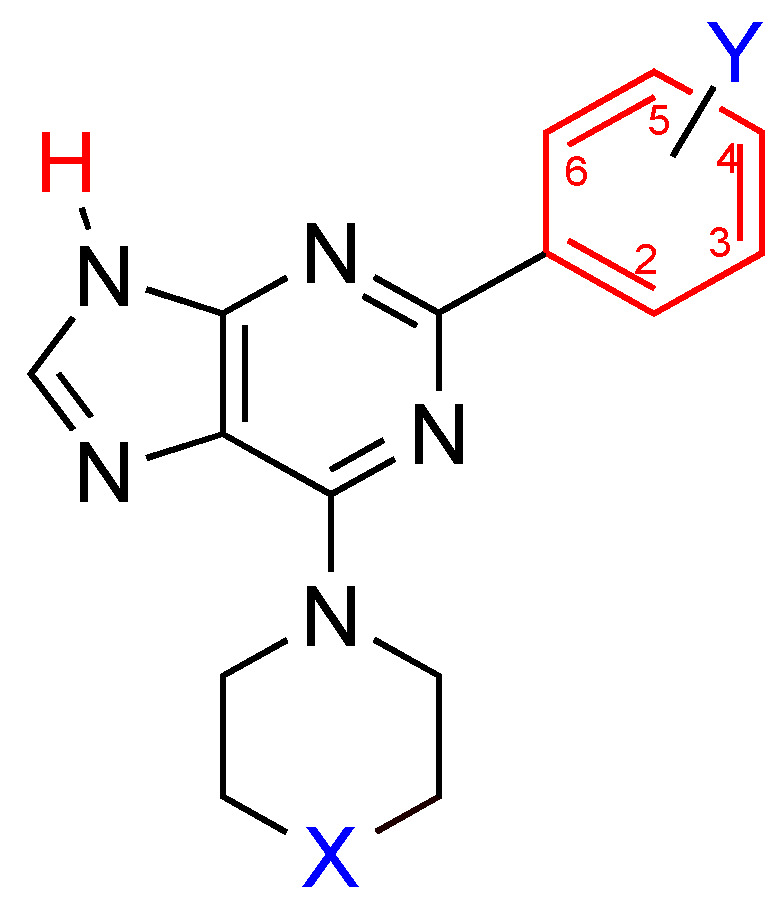
The adenosine receptor antagonists scaffold identified in our previous work.

In our previous work, we synthesized and identified a new scaffold for adenosine receptor antagonists based on the adenine nucleus (Figure 1) [28]. Highly potent and selective compounds were identified as having a piperidinyl group in C-6, a proton in N-9, and several selected aryl groups in C-2 of the purine nucleus. Indeed, the structure-activity relationship (SAR) study indicated that an aryl unit at C-2 is crucial for activity, and a proton at N-9 increased potency. Furthermore, a piperidinyl group in C-6 instead of a 4-*N*-methylpiperazinyl group led to purines with higher or similar affinity for the adenosine receptors A_1_ and A_3_ but with higher selectivity.

As an attempt to increase the affinity and selectivity of the compounds obtained in our previous work and to establish the importance of group X (Figure 1), herein, we synthesized new 6-morpholino purine derivatives substituted with a proton or a methyl group in N-9 and a selection of aryl substituents in the C-2 position of the purine nucleus. The results will allow us to complete the SAR study for the adenine-based scaffold identified in our previous publication.

## 2. Results and Discussion

### 2.1. Chemistry

**Scheme 1:**
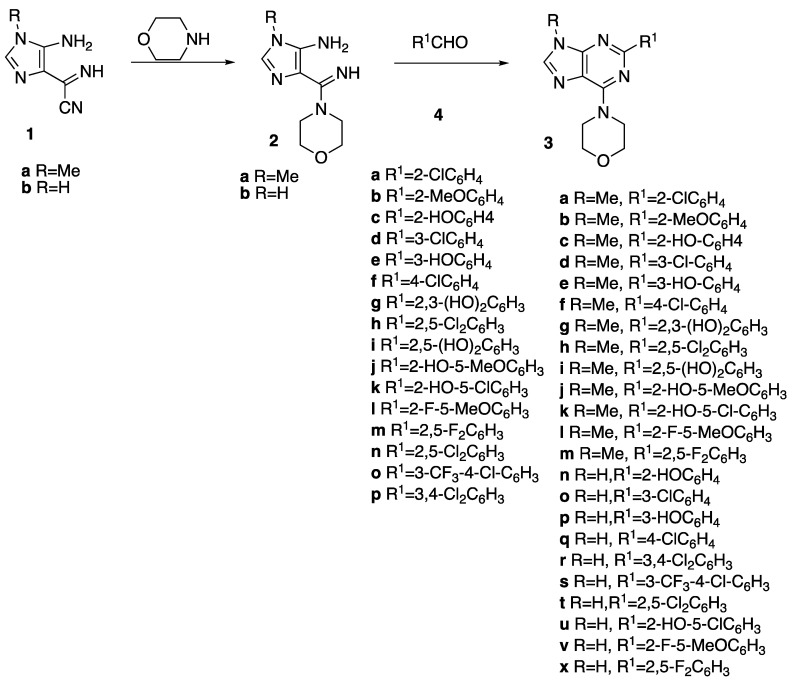
Synthetic approach to 2-aryl-adenine derivatives **3a–x**.

The target compounds **3a–x** were synthesized through the synthetic approach described in Scheme 1. The starting reagents **1** [29] and **2** [28,30] were obtained following the procedures described in previous work. Briefly, the commercially available diaminomaleonitrile was treated with triethyl orthoformate, under reflux. The solid obtained was reacted with the convenient amine followed by treatment with a base to generate imidazoles **1**. The compounds **2** were obtained in excellent yield through reaction of **1** with the excess of morpholine, in acetonitrile, at room temperature.

The products **3** were obtained through reaction of compounds **2** with different aldehydes **4** following a previously described methodology [28,30]. The experimental conditions used in each reaction mainly depended on the aldehyde. Generally, when imidazoles **1** were reacted with non-phenolic aldehydes **4**, the reactions were performed in basic medium. Considering that compounds **2** are temperature-sensitive, the reactions always started at low to moderate temperature. When the reactions were carried out under those conditions (Method A), they were slower, but the products **3** were precipitated pure from solution and isolated through simple filtration from the reaction medium. In order to accelerate the reactions, most of them started at moderate temperature, but, when the TLC showed the absence of imidazoles **2**, the temperature was increased and the reactions were monitored by TLC until the spots assigned to the intermediates were absent. In these cases, the reactions led to black-greyish solids that required purification. The purification was achieved through filtration of a dichloromethane solution of the compounds through a silica gel column.

To obtain the phenolic derivatives, the reactions were performed in an acidic medium until complete consumption of the reagents (evidenced by TLC) and then continued in a basic medium at a temperature between 40 and 80 °C. In these reactions, degradation of the reaction mixture also occurred when higher temperatures were used. The pure products were obtained following the purification procedure described above for the other derivatives. The new compounds were fully characterized by usual techniques (^1^H and ^13^C NMR spectra are presented in the supporting information).

### 2.2. Pharmacology

All compounds synthesized in this work have a morpholine group as a substituent at C-6, an H atom or a methyl group at N-9, and different substituted aryl groups at C-2 of the purine nucleus. The compounds (**3a–x**) were assessed through radioligand binding assays at all human adenosine subtype receptors (A_1_, A_2A_, A_2B_, and A_3_) expressed in mammalian cell lines. The percentage of inhibition (%_inhib_) of radioligand binding was determined for all compounds at 10 µM concentration. Those compounds showing a %_inhib_ higher than 80% were tested at different concentrations to determine their affinities (calculated as p*K*_i_) at the diverse receptors. Table 1 shows the binding affinities of all the synthesized compounds **3** at adenosine receptors A_1_, A_2A_, A_2B_, and A_3_. 

Analysis of the data presented in the Table 1 shows that at 10 µM, several compounds presented a percentage inhibition higher than 80%. The p*K_i_* was determined, being in the range of 5.28 ≤ p*K_i_* ≤ 8.23 (**3l**, **3x**). Both series, R = H and R = Me, gave ligands with high affinity to the four receptors subtypes; however, higher affinities are observed for derivatives with R = H (**3d** vs. **3h**, **3c** vs. **3n**, **3e** vs. **3p**, **3k** vs. **3u**, **3l** vs. **3v**, and **3m** vs. **3x**). The higher potency of derivatives with a proton at N-9 agrees with the results published in our previous work [28], but the increase in potency of N-9-methyl derivatives having a morpholine unit at C-6 may indicate that the morpholine ring is interacting with the target through the oxygen atom. When we look for selectivity, we find selective ligands either having a methyl group at N-9 (**3d**, **3e**, **3l** and **3m**) or a proton (**3p**, **3r**, **3s**, **3t** and **3v**). Additionally, the ligands with a methyl group at N-9 show high potency mainly for the A_1_ receptor (**3c**, **3d**, **3e**, **3i, 3j, 3k**, and **3m**), and ligands with a proton at N-9 presented high potency for both the A_1_ and A_3_ receptors (**3n**–**3x**). To evaluate the influence of the R^1^ group on potency and selectivity against the four receptors, we analyzed first the R^1^ = chloro-derivatives, compounds **3a**, **3d**, **3f**, **3h**, **3o**, **3q**, **3r**, **3s**, and **3t**. 

In this set, the most potent and selective compound for the A_1_ receptor was **3d**, with R^1^ = 3-ClC_6_H_4_ [p*K_i_* (A_1_) = 6.80 ± 0.07]. Compounds **3r** (R^1^ = 3,4-Cl_2_C_6_H_3_) and **3s** (R^1^ = 3-CF_3_-4-ClC_6_H_3_) were highly potent and selective for the A_3_ receptor. Compound **3q** (R^1^ = 4-ClC_6_H_4_) showed dual selectivity for A_1_/A_3_ receptors. These results seem to indicate that the presence of an electron-withdrawing chlorine atom at the *meta* position together with a hydrophobic methyl group at N-9 interacts efficiently with the A_1_ receptor (**3d**). However, when the hydrophilic hydrogen was at N-9, a promiscuous ligand resulted (**3o**). The presence of a chlorine atom at the *para* position and a proton at N-9 interacts efficiently with receptors A_1_ and A_3_ (**3q**). Compound **3q** is a potential dual ligand for A_1_/A_3_ receptors with therapeutic interest, as A_1_ and A_3_ receptors, principally coupled to G_i/o_ proteins, can simultaneously inhibit the adenylyl cyclase activity [6,31,32].

In addition, selectivity was achieved when a second chlorine or a CF_3_ group was simultaneously at the *meta* position (**3r**, **3s**), suggesting volume constraints at the A_1_, A_2A_, and A_2B_ pockets interacting with the ligands’ *meta* position. This hypothesis is also supported by the lower p*K_i_* observed for compound **3s** (R^1^ = 3-CF_3_-4-Cl-C_6_H_3_; p*K_i_* = 6.02 ± 0.04) when compared to the p*K_i_* of compound **3r** (R^1^ = 3,4-Cl_2_C_6_H_3_; p*K_i_* = 7.15 ± 0.08). 

The sub-set of derivatives with hydroxyl groups at *ortho* or *meta* positions of the R^1^ group (**3c**, **3e**, **3g**, **3i**, **3j**, **3k**, **3n**, **3p**, and **3u**) will be analyzed. Generally, a hydroxyl group at *ortho* or *meta* positions of R^1^ led to very potent but not selective ligands (**3c**, **3i**, **3j**, **3k**, **3n**, and **3u**), except compounds **3e** and **3p**, which have a hydroxyl group at the *meta* position of R^1^ and showed selectivity for the A_1_ receptor. Derivative **3d** (R^1^ = 3-ClC_6_H_4_) also showed selectivity for the A_1_ receptor. These results suggest that an electron-withdrawing group produced through the inductive effect at the *meta* position of R^1^ interacts efficiently with a polar moiety at the A_1_ receptor. Comparing the affinity values between **3b** (R^1^ = 2-MeOC_6_H_4_) and **3c** (R^1^ = 2-HOC_6_H_4_), the increase of affinity of **3c** suggests that a hydrogen-bond donor (HBD) group at the *ortho* position of R^1^ is important for the interaction with the adenosine receptors A_1_, A_2A_, and A_2B_. However, the possibility of a hydrogen-bond-acceptor (HBA) interaction of the ligand with the target should also be considered. Hydroxyl and methoxyl groups may interact that way with the targets; however, if the receptor pockets have volume constraints, this will explain the lack of activity of derivative **3b** and support the hypothesis of HBD interaction. Furthermore, compound **3c** has almost the same submicromolar affinity for the A_1_ and A_2A_ receptors. This result indicates that **3c** is a dual ligand for A_1_/A_2A_ receptors. Dual affinity for the A_1_/A_2A_ receptors was also observed for ligand **2k**. These ligands could have medicinal interest, as compounds with dual affinity at A_1_/A_2A_ receptors have shown therapeutic efficacy in animal models of Parkinson’s disease [33,34]. On the other hand, compound **3u** showed a dual affinity for receptors A_1_/A_2B_. A comparison of affinities registered for **3c**, **3d**, and **3e** with **3n**, **3o**, and **3p** suggests more hydrophilic or small pockets at receptors to accommodate the group at N-9 of the ligands. Additionally, compound **3e** (R^1^ = 3-HOC_6_H_4_) is a very potent and selective ligand for the A_1_ receptor with p*K_i_* = 6.16 ± 0.21, while compound **3g** (R^1^ = 2,3-(HO)_2_C_6_H_3_) has a low affinity for all of the receptors. This may indicate that an intramolecular hydrogen bond between the hydroxyl groups of the ligand precludes the interaction with the target, leading to low activity.

**Figure 2:**
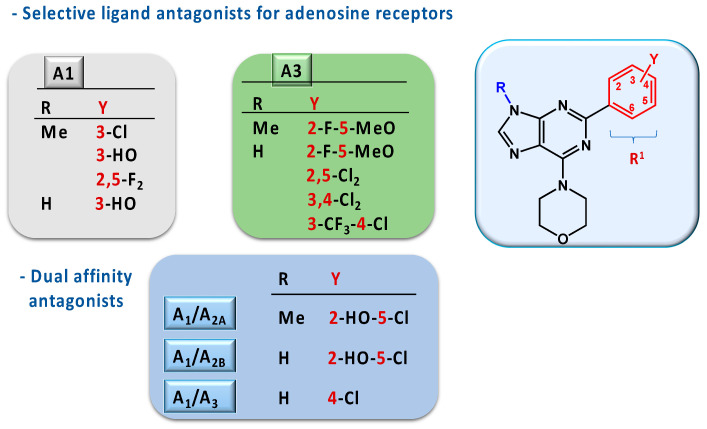
Structure-activity relationship of 2-aryl-9-substituted-6-morpholino purine derivatives for the adenosine receptor antagonists.

Finally, the fluoro derivatives (**3l**, **3m**, **3v**, and **3x**) presented high potency and selectivity for receptors A_1_ and A_3_, except compound **3x**, which showed no selectivity. Compounds **3l** and **3v** with R^1^ = 2-F-5-MeOC_6_H_3_ were selective to the A_3_ receptor, with p*K_i_* = 5.28 ± 0.11 and p*K_i_* = 7.83 ± 0.16, respectively. Compound **3v** also showed a high affinity for receptor A_2B_ (p*K_i_* = 6.40 ± 0.16). These results suggest volume restrictions in the pockets of receptors A_2B_ and A_3_ to lodge the methyl group at N-9 of the ligand, with the available space in receptor A_3_ bigger than in receptor A_2B_. Compound **3m** showed high affinity and selectivity for the A_1_ receptor, while compound **3x** showed higher potency for all receptors but no selectivity. These results also support the hypothesis that there is limited space in the receptors’ pockets to accommodate bulky groups at N-9. The difference in the selectivity of compounds **3l** and **3m** may indicate that receptor A_3_ has a hydrophobic pocket to accommodate the substituent MeO present at the *meta* position of the ligand, while receptor A_1_ does not. This hypothesis is also supported by the results obtained for ligands **3v** and **3x**. Figure 2 represents a SAR model that summarizes the results.

**Figure 3:**
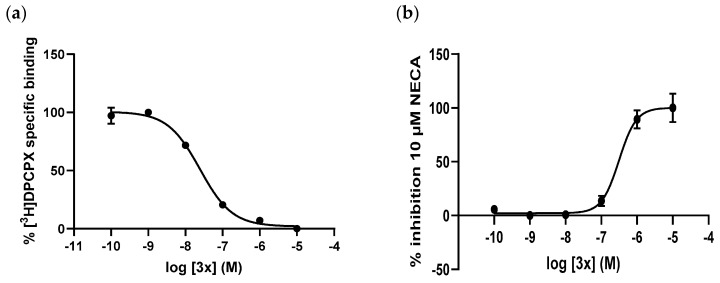
(**a**) Competition binding curve for compound **3x** at A_1_ receptors. (**b**) Concentration-response curve of compound **3x** in the presence of 10 µM of NECA at human A_1_ receptor expressed in CHO cells. Points represent the mean ± standard deviation (vertical bars) of duplicate measurements.

The most potent compounds for A_1_ (**3x**; p*K_i_* = 8.23 ± 0.06; Figure 3a) and A_3_ (**3v**; p*K_i_* = 7.83 ± 0.16) receptors were selected and tested in intracellular cAMP tests to study their functional activity. The agonist/antagonist behavior of the two compounds selected was determined by assessing their effect on the modulation of cAMP levels by the receptor agonist NECA. Figure 3b shows the result of a representative test for the antagonist potency of compound **3x** at A_1_ receptor. The results confirmed that the compounds **3v** and **3x** are antagonists of the A_3_ and A_1_ receptors, and their antagonist potencies, expressed as p*K_B_* values, were 8.24 ± 0.11 and 8.25 ± 0.16, respectively (Table 2). Based on their structural similarity, we can generalize that all compounds of this work are antagonists of adenosine receptors.

In this work, we synthesized highly potent and selective hits based on the scaffold for adenosine receptor antagonists identified in our previous publication [28]. These results indicate that the purines substituted with a morpholine group at C-6, a hydrogen atom or a methyl group at N-9, and a specific aryl group at C-2 of purine nucleus, gave high affinity and selective antagonists for adenosine A_1_ and A_3_ receptors, as well as dual antagonists for receptors A_1_/A_2A_, A_1_/A_2B_, and A_1_/A_3_. In addition, four compounds (**3i**, **3j**, **3n**, and **3o**) showed high affinity, although they were not selective for any specific adenosine receptor subtype.

### 2.3. Docking Studies 

**Figure 4:**
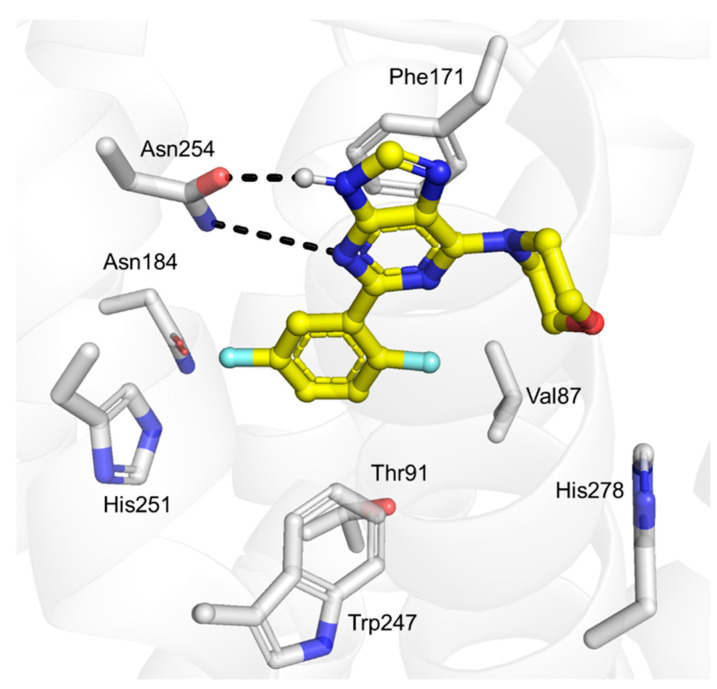
Predicted binding mode of compound **3x** in the orthosteric site of the A_1_ receptor. The receptor is depicted as a grey cartoon, with the ligand and key side chains represented as sticks. Hydrogen bonds are shown as black dashed lines.

Compound **3x** was computationally docked to the orthosteric site of the A_1_ receptor using a crystal structure representing an inactive conformation (PDB accession code: 5UEN) [35]. The ligand was predicted to form hydrogen bonds with Asn254 and π-π stacking with Phe171, which are interactions characteristic of adenosine receptor antagonists (Figure 4) [36]. The predicted binding mode of compound **3x** in the A_1_ receptor also explained observed SARs. For example, replacing the fluorine at the 5-position of the phenyl ring with a methoxy group led to a substantial loss of affinity at the A_1_ receptor (p*K_i_* < 5, compound **3v**). Consistent with this observation, increasing the size of this substituent in our model of the complex led to clashes with Asn184 and His251.

## 3. Experimental Protocols

### 3.1. Chemistry

The imidazoles **1** used in this work were synthesized according to previously described procedures [29], and compounds **2** were synthesized according to the procedure described in [28,30]. Solvents and other commercially available chemicals were used as shipped. The melting points (m.p.) were determined with a Gallenkamp melting point apparatus, and they are uncorrected. The reactions were followed by thin layer chromatography (TLC) using Silica Gel 60 F_254_ (Merck, Darmstadt, Germany) plates with detection by UV light. The NMR spectra were obtained on a Varian Unity Plus (^1^H: 300 MHz, ^13^C: 75 MHz) or on a Bruker Avance III NMR spectrometer (^1^H: 400 MHz, ^13^C: 100 MHz) including the ^1^H and ^13^C bidimensional correlation spectra (HMQC and HMBC). For solutions, [D_6_]-DMSO residual [D_6_]-DMSO (*δ*_H_ = 2.49 ppm) or [D_6_]-DMSO (*δ*_C_ = 39.5 ppm) was used as the internal standard at 298 K. Chemical shifts (*δ*) were reported in parts per million (ppm) and the coupling constants, *J*, were presented in hertz (Hz). The purity of all tested compounds was higher than 95% according to elemental analysis, which was reported to be within 0.4% of the calculated values. The IR spectra were recorded with a FT-IR Bomem MB 104 using nujol mulls and NaCl cells. Elemental analyses were performed with a LECO CHNS-932 instrument.

### 3.2. General Procedure for the Synthesis of **3a–x**

**Method A**: To a suspension of imidazole **2** in ethanol, an ethanol/acetonitrile mixture, or DMSO, the aldehyde **4** (1.1–1.5 equivalents) was added, followed by triethylamine (10 equivalents). The reaction was kept at room to moderate temperature and followed by TLC. Otherwise, when the TLC indicated the absence of the starting reagent but the presence of intermediates, the reaction continued at a higher temperature. When the TLC showed the absence of intermediate spots, the solvent was removed in the rotary evaporator, and an off-white solid was isolated after the addition of a small amount of ethanol to the residue. When the residue showed a dark color, it was dissolved in DCM. The solution was filtered through a column of silica gel (0.5 cm high), and the column was washed with an extra 30 mL of DCM. The resulting solution was concentrated until dry, diethyl ether was added to the oil, and an off-white solid was precipitated. The solid was filtered and washed with cold diethyl ether. 

**Method B**: To a suspension of imidazole **2** in ethanol, the aldehyde **4** (1.1–1.5 equivalents) was added, followed by trifluoroacetic acid (1.3–2.0 equivalents). The reaction was kept at 22 °C under a magnetic stirrer until the TLC showed consumption of starting reagent **2**. The solvent was eliminated in the rotary evaporator, dimethyl sulfoxide and/or ethanol was added, followed by triethylamine (10 equivalents), and the reaction continued at 40–80 °C. When the TLC indicated the end of the reaction, the solvents were removed in the rotary evaporator, and the product was precipitated through the addition of water. The isolated solid was dissolved in a mixture of DCM/THF/EtOH and purified through dry flash chromatography using DCM as the elution solvent.

#### 3.2.1. 4-(2-(2-Chlorophenyl)-9-methyl-9H-purin-6-yl)morpholine **3a**

Method A: Compound **2a** (0.18 g, 0.89 mmol) in EtOH, aldehyde **4a** (1.1 eq.), and Et_3_N (10 eq.) was kept at 22 °C for 20 days. Product **3a** was isolated as an off-white solid (0.12 g, 0.28 mmol, 31%); m.p. = 99–101 °C; Found: C, 58.28; H, 4.86; N, 21.25. C_16_H_16_ClN_5_O requires C, 58.27; H, 4.89; N, 21.24; IR (Nujol mull) νmax: 1584 cm^−1^; ^1^H (300 MHz, DMSO-d6) *δ*: 8.21 (s, 1H), 7.75–7.72 (m, 1H), 7.53–7.50 (m, 1H), 7.45–7.38 (m, 2H), 4.23 (br s, 4H), 3.75 (s, 3H), 3.71 (t, *J* = 4.5 Hz, 4H); ^13^C (75 MHz, DMSO-d6) *δ*: 157.6, 152.7, 151.6, 141.4, 138.7, 131.6, 131.5, 130.1, 129.9, 126.8, 117.7, 66.2, 45.2, 29.6.

#### 3.2.2. 4-(2-(2-Methoxyphenyl)-9-methyl-9H-purin-6-yl)morpholine **3b**

Method A: Compound **2a** (0.13 g, 0.64 mmol) in DMSO, aldehyde **4b** (1.1 eq.), and Et_3_N (10 eq.) was kept at 60 °C for 6 days. Product **3b** was isolated as an off-white solid (0.05 g, 0.23 mmol, 36%); m.p. = 114–116 °C; Found: C, 62.10; H, 5.92; N, 21.15. C_17_H_19_N_5_O_2_.0.2H_2_O requires C, 62.08; H, 5.90; N, 21.30; IR (Nujol mull) νmax: 1698, 1587, 1577 cm^−1^; ^1^H (300 MHz, DMSO-d6) *δ*: 8.15 (s, 1H), 7.50 (dd, *J* = 7.7 Hz, *J* = 1.8 Hz, 1H), 7.37 (td, *J* = 7.8 Hz, *J* = 1.8 Hz, 1H), 7.09 (d, *J* = 7.8 Hz, 1H), 6.99 (t, *J* = 7.8 Hz, 1H), 4.20 (br s, 4H), 3.80–3.50 (m, 10H). ^13^C (75 MHz, DMSO-d6) *δ*: 158.4, 157.2, 152.8, 151.7, 149.9, 130.6, 130.1, 129.7, 120.0, 112.5, 66.2, 55.9, 45.2, 29.5. 

#### 3.2.3. 2-(9-Methyl-6-morpholino-9H-purin-2-yl)phenol **3c**

Method A: Compound **2a** (0.08 g, 0.38 mmol) in a mixture of EtOH/CH_3_CN (1/1) and aldehyde **4c** (1.5 eq.) was kept at 11 °C for 17 days. Product **3c** was isolated as an off-white solid (0.08 g, 0.25 mmol, 66%); m.p. = 210–212 °C (Found: C, 61.17; H, 5.52; N, 21.00. C_16_H_17_N_5_O_2_.0.1H_2_O requires C, 61.45; H, 5.49; N, 22.40); IR (Nujol mull) νmax: 3097, 1591, 1530, 1519 cm^−1^; ^1^H (300 MHz, DMSO-d6) *δ*: 13.52 (s, 1H, OH), 8.38 (dd, *J* = 8.4 Hz, *J* = 1.8 Hz, 1H), 8.21 (s, 1H), 7.33 (td, *J* = 7.8 Hz, *J* = 1.8 Hz, 1H), 6.91 (t, *J* = 7.8 Hz, 1H), 6.89 (d, *J* = 7.8 Hz, 1H), 4.25 (br s, 4H), 3.78 (s, 4H), 3.77 (s, 3H); ^13^C (75 MHz, DMSO-d6) *δ*: 159.4, 157.5, 152.4, 149.7, 141.4, 132.0, 129.0, 119.3, 118.6, 117.8, 117.2, 66.1, 45.4, 29.6.

#### 3.2.4. 4-(2-(3-Chlorophenyl)-9-methyl-9H-purin-6-yl)morpholine **3d**

Method A: Compound **2a** (0.31 g, 1.46 mmol) in EtOH, aldehyde **4d** (1.1 eq.), and Et_3_N (10 eq.) was kept at 20 °C for 12 days and then at 70 °C for 21 h. The product **3d** was isolated as an off-white solid (0.35 g, 1.06 mmol, 73%); m.p. = 160–162 °C (Found: C, 58.04; H, 4.76; N, 21.18. C_16_H_16_ClN_5_O requires C, 58.27; H, 4.89; N, 21.24); IR (Nujol mull) νmax: 1690, 1575 cm^−1^; ^1^H (300 MHz, DMSO-d6) *δ*: 8.31 (m, 2H), 8.17 (s, 1H), 7.48–7.50 (m, 2H), 4.26 (br s, 4H), 3.76 (br s, 4H), 3.73 (s, 3H); ^13^C (75 MHz, DMSO-d6) *δ*: 155.3, 153.0, 151.9, 141.5, 140.4, 133.2, 130.2, 129.6, 127.1, 126.3, 118.4, 66.2, 45.3, 29.5.

#### 3.2.5. 3-(9-Methyl-6-morpholino-9H-purin-2-yl)phenol **3e**

Method B: Compound **2b** (0.22 g, 1.10 mmol) in EtOH, aldehyde **4e** (1.1 eq.), and TFA (2 eq.) was kept at 22 °C for 3 days. Then, the reaction continued in DMSO and Et_3_N (10 eq.) at 60 °C for 2 days. The product **3e** was isolated as an off-white solid (0.23 g, 0.75 mmol, 67%); m.p. = 230–232 °C (Found: C, 61.67; H, 5.52; N, 22.64. C_16_H_17_N_5_O_2_ requires C, 61.72; H, 5.50; N, 22.49); IR (Nujol mull) νmax: 3281, 1578, 1505 cm^−1^; ^1^H (300 MHz, DMSO-d6) *δ*: 9.45 (s, 1H, OH), 8.16 (s, 1H), 7.84 (br.s, 2H), 7.24 (t, *J* = 8.4 Hz, 1H), 6.82 (d, *J* = 7.8 Hz, 1H), 4.28 (br s, 4H), 3.77 (s, 3H), 3.75 (m, 4H); ^13^C (75 MHz, DMSO-d6) *δ*: 157.3, 156.8, 152.9, 152.0, 141.1, 139.7, 129.1, 118.6, 118.1, 116.7, 114.5, 66.2, 45.1, 29.4. 

#### 3.2.6. 4-(2-(4-Chlorophenyl)-9-methyl-9H-purin-6-yl)morpholine **3f**

Method A: Compound **2a** (0.34 g, 1.57 mmol) in EtOH, aldehyde **4f** (1.1 eq.), and Et_3_N (10 eq.) was kept at 21 °C for 8 days and then at 70 °C for 6 h. The product **3f** was isolated as an off-white solid (0.25 g, 0.75 mmol, 48%); m.p. = 226–228 °C (Found: C, 57.99; H, 4.86; N, 21.13. C_16_H_16_ClN_5_O requires: C, 58.27; H, 4.89; N, 21.24); IR (Nujol mull) νmax: 1706, 1587, 1571 cm^−1^; ^1^H (300 MHz, DMSO-d6) *δ*: 8.40 (d, *J* = 8.4 Hz, 2H), 8.17 (s, 1H), 7.52 (d, *J* = 8.4 Hz, 2H), 4.28 (br s, 4H), 3.79 (s, 3H), 3.74 (m, 4H); ^13^C (75 MHz, DMSO-d6) *δ*: 155.7, 152.9, 151.0, 141.4, 137.1, 134.5, 129.4, 128.3, 118.2, 66.2, 45.1, 29.5.

#### 3.2.7. 3-(9-Methyl-6-morpholino-9H-purin-2-yl)benzene-1,2-diol **3g**

Method B: Compound **2a** (0.15 g, 0.69 mmol) in EtOH, aldehyde **4g** (1.1 eq.), and TFA (2 eq.) was kept at 22 °C for 3 days. Then, the reaction continued in a mixture of DMSO/EtOH (0.1/1) and Et_3_N (10 eq.) at 80 °C for 6 h. The product **3g** was isolated as an off-white solid (0.14 g, 0.43 mmol, 62%); m.p. = 254–256 °C (Found: C, 58.87; H, 5.55; N, 21.02. C_16_H_17_N_5_O_3_ C16H17N5O3 requires C, 58.71; H, 5.23; N, 21.39); IR (Nujol mull) νmax: 3415, 1588, 1569 cm^−1^; ^1^H (300 MHz, DMSO-d6) *δ*: 13.73 (s, 1H, OH), 8.83 (br s, 1H, OH), 8.19 (s, 1H), 7.83 (dd, *J* = 8.0 Hz, *J* = 1.5 Hz, 1H), 6.84 (dd, *J* = 7.5 Hz, *J* = 1.5 Hz, 1H), 6.70 (t, *J* = 7.8 Hz, 1H), 4.23 (br s, 4H), 3.77 t, *J* = 5.4 Hz, 7H); ^13^C (75 MHz, DMSO-d6) *δ*: 157.9, 152.3, 149.8, 148.3, 146.1, 141.3, 119.5, 119.1, 117.9, 117.6, 117.3, 66.0, 45.4.

#### 3.2.8. 4-(2-(2,5-Dichlorophenyl)-9-methyl-9H-purin-6-yl)morpholine **3h**

Method A: Compound **2a** (0.25 g, 1.22 mmol) in EtOH, aldehyde **4h** (1.1 eq.), and Et_3_N (10 eq.) was kept at 40 °C for 4 days and then at 50 °C for 10 days. The product **3h** was isolated as an off-white solid (0.18 g, 0.50 mmol, 41%); m.p. = 141–142 °C (Found: C, 52.64; H, 4.11; N, 19.45. C_16_H_15_Cl_2_N_5_O requires C, 52.76; H, 4.15; N, 19.23); IR (Nujol mull) νmax: 3048, 1585 cm^−1^; ^1^H (300 MHz, DMSO-d6) *δ*: 8.22 (s, 1H), 7.78(d, *J* = 2.7 Hz, 1H), 7.56 (d, *J* = 8.4 Hz, 1H), 7.50 (dd, *J* = 8.4 Hz, *J* = 2.4 Hz, 1H), 4.23 (br s, 4H), 3.76 (s, 3H), 3.72 (t, *J* = 4.5 Hz, 4H); ^13^C (75 MHz, DMSO-d6) *δ*: 156.3, 152.7, 151.5, 141.6, 140.0, 131.9, 131.4, 130.9, 130.5, 129.7, 117.9, 66.2, 45.2, 29.6.

#### 3.2.9. 2-(9-Methyl-6-morpholino-9H-purin-2-yl)benzene-1,4-diol **3i**

Method B: Compound **2a** (0.20 g, 0.96 mmol) in EtOH, aldehyde **4i** (1.1 eq.), and TFA (2 eq.) was kept at 22 °C for 5 h. Then, the reaction continued in a mixture of DMSO/EtOH (0.1/1) and Et_3_N (10 eq.) at 60 °C for 3 days. The product **3i** was isolated as an off-white solid (0.20 g, 0.61 mmol, 64%); m.p. = 288–290 °C (Found: C, 57.21; H, 5.09; N, 20.61. C_16_H_17_N_5_O_3_.0.5H_2_O requires C, 57.14; H, 5.36; N, 20.83); IR (Nujol mull) νmax: 3353, 1574, 1500 cm^−1^; ^1^H (300 MHz, DMSO-d6) *δ*: 12.81 (s, 1H, OH), 8.88 (s, 1H, OH), 8.20 (s, 1H), 7.80 (d, *J* = 2.7 Hz, 1H), 6.77 (dd, *J* = 8.6 Hz, *J* = 2.7 Hz, 1H), 6.72 (d, *J* = 8.7 Hz, 1H), 4.25 (br s, 4H), 3.77 (t, *J* = 3.6 Hz, 7H); ^13^C (75 MHz, DMSO-d6) *δ*: 157.6, 152.4, 152.2, 149.9, 149.2, 141.4, 119.8, 119.3, 117.7 (2C), 114.1, 66.1, 45.4, 29.7.

#### 3.2.10. 4-Methoxy-2-(9-methyl-6-morpholino-9H-purin-2-yl)phenol **3j**

Method B: Compound **2a** (0.15 g, 0.70 mmol) in EtOH, aldehyde **4j** (1.2 eq.), and TFA (2 eq.) was kept at 22 °C for 2 days. Then, the reaction continued in EtOH and Et_3_N (10 eq.) at 25 °C for 5 days. The product **3j** was isolated as an off-white solid (0.14 g, 0.43 mmol, 62%); m.p. = 146–148 °C (Found: C, 59.75; H, 5.67; N, 20.42. C_17_H_19_N_5_O_3_ requires C, 59.81; H, 5.61; N, 20.52); IR (Nujol mull) νmax: 3011, 1594, 1578 cm^−1^; ^1^H (300 MHz, DMSO-d6) *δ*: 12.99 (s, 1H, OH), 8.20 (s, 1H), 7.89 (d, *J* = 3.2 Hz, 1H), 6.97 (dd, *J* = 8.8 Hz, *J* = 3.2 Hz, 1H), 6.83 (d, *J* = 8.8 Hz, 1H), 4.23 (s, 4H), 377 (m, 7H); ^13^C (75 MHz, DMSO-d6) *δ*: 157.2, 153.4, 152.4, 151.5, 149.8, 141.4, 119.3, 118.7, 117.9, 117.8, 112.6, 66.0, 55.4, 45.4, 29.6.

#### 3.2.11. 4-Chloro-2-(9-methyl-6-morpholino-9H-purin-2-yl)phenol **3k**

Method B: Compound **2a** (0.13 g, 0.64 mmol) in EtOH, aldehyde **4k** (1.1 eq.), and TFA (2 eq.) was kept at 22 °C for 2 days. Then, the reaction continued in a mixture of DMSO/EtOH (0.2/1) and Et_3_N (10 eq.) at 60 °C for 6 days. The product **3k** was isolated as an off-white solid (0.104 g, 0.41 mmol, 64%); m.p. = 179–180 °C (Found: C, 55.65; H, 4.50; N, 20.00. C_16_H_16_ClN_5_O_2_ requires C, 55.58; H, 4.66; N, 20.25); IR (Nujol mull) νmax: 3016, 1589, 1528 cm^−1^; ^1^H (300 MHz, DMSO-d6) *δ*: 13.59 (s, 1H, OH), 8.24 (d, *J* = 2.8 Hz, 1H), 8.20 (s, 1H), 7.32 (dd, *J* = 8.8 Hz, *J* = 2.8 Hz, 1H), 6.90 (d, *J* = 8.8 Hz, 1H), 4.20 (s, 4H), 3.77 (t, *J* = 4.8 Hz, 7H); ^13^C (75 MHz, DMSO-d6) *δ*: 158.0, 156.0, 152.2, 149.5, 141.5, 131.4, 127.7, 122.2, 120.5, 119.1, 118.0, 66.0, 45.3, 29.6.

#### 3.2.12. 4-(2-(2-Fluoro-5-methoxyphenyl)-9-methyl-9H-purin-6-yl)morpholine **3l**

Method A: Compound **2a** (0.16 g, 0.77 mmol) in EtOH, aldehyde **4l** (1.3 eq.), and Et_3_N (10 eq.) was kept at 22 °C for 1 day and then at 60 °C for 4 days. The product **3l** was isolated as an off-white solid (0.15 g, 0.44mmol, 57%); m.p. = 102–104 °C (Found: C, 58.76; H, 4.93; N, 19.99. C_17_H_18_FN_5_O_2_·0.2H_2_O requires C, 58.86; H, 5.31; N, 20.19); IR (Nujol mull) νmax: 3122, 1598, 1579 cm^−1^; ^1^H (300 MHz, DMSO-d6) *δ*: 8.19 (s, 1H), 7.52 (dd, *J* = 3.2 Hz, *J* = 6.4 Hz, 1H), 7.18 (dd, *J* = 8.0 Hz, *J* = 6.9 Hz, 1H), 7.01 (dt, *J* = 6.9 Hz, *J* = 2.4 Hz, 1H), 4.23 (br s, 4H), 3.80–3.70 (m, 10H). ^13^C (75 MHz, DMSO-d6) *δ*: 155.2 (d, *J* = 4 Hz), 154.9 (d, *J* = 2 Hz), 154.7 (d, *J* = 244 Hz), 153.4, 151.7, 141.4, 127.7 (d, *J* = 11 Hz), 117.8, 117.3 (d, *J* = 24 Hz), 115.9 (d, *J* = 2 Hz), 115.8 (d, *J* = 8 Hz), 66.2, 55.6, 45.2, 29.5.

#### 3.2.13. 4-(2-(2,5-Difluorophenyl)-9-methyl-9H-purin-6-yl)morpholine **3m**

Method A: Compound **2a** (0.31 g, 1.48 mmol) in EtOH, aldehyde **4m** (1.1 eq.), and Et_3_N (10 eq.) was kept at 40 °C for 4 days and then at 50 °C for 6 days. The product **3m** was isolated as an off-white solid (0.30 g, 0.91 mmol, 61%); m.p. = 143–145 °C (Found: C, 57.90; H, 4.77; N, 20.97. C_16_H_15_F_2_N_5_O requires C, 58.00; H, 4.56; N, 21.14); IR (Nujol mull) νmax: 3083, 1616, 1578 cm^−1^; ^1^H (400 MHz, DMSO-d6) *δ*: 8.20 (s, 1H), 7.80–7.79 (m, 1H), 7.34–7.30 (m, 1H), 4.24 (br s, 4H), 3.77 (s, 3H), 3.73 (t, *J* = 4.5 Hz, 4H); ^13^C (100 MHz, DMSO-d6) *δ*: 157.7 (dd, *J* = 230 Hz, *J* = 6 Hz), 156.5 (dd, *J* = 246 Hz, *J* = 4 Hz), 154.2 (dd, *J* = 5 Hz, *J* = 2 Hz), 152.9, 151.6, 141.6, 128.5 (dd, *J* = 11 Hz, *J* = 4 Hz), 118.4 (dd, *J* = 25 Hz, *J* = 9 Hz), 118.0, 117.5 (dd, *J* = 24 Hz, *J* = 9 Hz), 128.3 (dd, *J* = 25 Hz, *J* = 2 Hz), 66.2, 45.2, 29.5.

#### 3.2.14. 2-(6-Morpholino-9H-purin-2-yl)phenol **3n**

Method B: Compound **2b** (0.30 g, 1.55 mmol) in EtOH, aldehyde **4c** (1.1 eq.), and TFA (1.5 eq.) was kept at 22 °C for 4.5 h. Then, the reaction continued in DMSO and Et_3_N (10 eq.) at 40 °C for 10 days. The product **3n** was isolated as an off-white solid (0.15 g, 0.52 mmol, 34%); (Found: C, 60.43; H, 5.12; N, 23.71. C_15_H_15_N_5_O_2_ requires C, 60.60; H, 5.09; N, 23.56); ^1^H (400 MHz, DMSO-d6) *δ*: 13.64 (s, 1H), 13.23 (br s, 1H), 8.35 (dd, *J* = 8.4 Hz, *J* = 2.0 Hz, 1H), 8.20 (s, 1H), 7.31 (dt, *J* = 8.4 Hz, *J* = 2.0 Hz, 1H), 6.90 (m, 2H), 4.28 (br s, 4H), 3.78 (m, 4H); ^13^C (100 MHz, DMSO-d6) *δ*: 159.4, 157.5, 152.5, 149.9, 139.0, 131.8, 128.9, 119.4, 118.6, 117.6, 117.2, 66.1, 45.5.

#### 3.2.15. 4-(2-(3-Chlorophenyl)-9H-purin-6-yl)morpholine **3o**

Method A: Compound **2b** (0.14 g, 0.72 mmol) in EtOH, aldehyde **4d** (1.1 eq.), and Et_3_N (10 eq.) was kept at 40 °C for 10 days. The product **3o** was isolated as an off-white solid (0.10 g, 0.32 mmol, 44%); m.p. = 265–267 °C (Found: C, 56.21; H, 4.26; N, 21.66. C_15_H_14_ClN_5_O.0.3H_2_O requires C, 56.10; H, 4.55; N, 21.81); IR (Nujol mull) νmax: 3103, 1587, 1571 cm^−1^; ^1^H (300 MHz, DMSO-d6) *δ*: 13.16 (s, 1H, NH), 8.18 (s, 1H), 8.55–7.48 (m, 2H), 8.32–8.28 (m, 2H), 4.29 (br s, 4H), 3.76 (t, *J* = 4.8 Hz, 4H); ^13^C (75 MHz, DMSO-d6) *δ*: 155.2, 152.9, 152.7, 140.7, 139.4, 133.1, 130.2, 129.3, 127.0, 126.1, 118.3, 66.2, 45.1.

#### 3.2.16. 3-(6-Morpholino-9H-purin-2-yl)phenol **3p**

Method B: Compound **2a** (0.31 g, 1.58 mmol) in EtOH, aldehyde **4e** (1.1 eq.), and TFA (1.5 eq.) was kept at 22 °C for 7 days. Then, the reaction continued in DMSO and Et_3_N (10 eq.) at 50 °C for 1 month. The product **3p** was isolated as an off-white solid (0.24 g, 0.81 mmol, 51%); (Found: C, 60.53; H, 5.02; N, 23.52. C_15_H_15_N_5_O_2_ requires C, 60.60; H, 5.09; N, 23.56); ^1^H (300 MHz, DMSO-d6) *δ*: 13.07 (br s, 1H), 9.45 (br s, 1H), 8.13 (s, 1H), 7.77–7.79 (m, 2H), 7.23 (t, *J* = 7.8 Hz, 1H), 6.82 (ddd, *J* = 7.8 Hz, *J* = 3.3 Hz, *J* = 0.9 Hz, 1H), 4.28 (br s, 4H), 3.76 (m, 4H); ^13^C (75 MHz, DMSO-d6 *δ*: 157.3, 156.9, 152.9, 152.6, 139.9, 138.8, 129.2, 118.5, 117.9, 116.6, 114.4, 66.3, 45.1.

#### 3.2.17. 4-(2-(4-Chlorophenyl)-9H-purin-6-yl)morpholine **3q**

Method A: Compound **2b** (0.31 g, 1.58 mmol) in EtOH, aldehyde **4f** (1.1 eq.), and Et_3_N (10 eq.) was kept at 20 °C for 13 days and then at 50 °C for 21 h. The product **3q** was isolated as an off-white solid (0.15 g, 0.47 mmol, 30%); m.p. = 284 °C (dec.) (Found: C, 57.00; H, 4.51; N, 22.05. C_15_H_14_ClN_5_O requires C, 57.06; H, 4.47; N, 22.18); IR (Nujol mull) νmax: 1583 cm^−1^; ^1^H (300 MHz, DMSO-d6) *δ*: 13.13 (s, 1H), 8.34 (s, 1H), 8.16 (d, *J* = 9.0 Hz, 2H), 7.50 (d, *J* = 9.0 Hz, 2H), 4.28 (br s, 4H), 3.75 (m, 4H); ^13^C (75 MHz, DMSO-d6) *δ*: 155.8, 153.0, 152.5, 139.0, 137.3, 134.4, 129.3, 128.3, 118.1, 66.2, 45.3.

#### 3.2.18. 4-(2-(3,4-Dichlorophenyl)-9H-purin-6-yl)morpholine **3r**

Method A: Compound **2b** (0.31 g, 1.57 mmol) in EtOH, aldehyde **4p** (1.1 eq.), and Et_3_N (10 eq.) was kept at 40 °C for 2 days and then at 50 °C for 6 days. The product **3t** was isolated as an off-white solid (0.18 g, 0.51 mmol, 33%); m.p. > 300 °C (Found: C, 51.28; H, 3.91; N, 19.93. C_15_H_13_Cl_2_N_5_O requires C, 51.45; H, 3.74; N, 20.00); IR (Nujol mull) νmax: 3010, 1606, 1584, 1561 cm^−1^; ^1^H (300 MHz, DMSO-d6) *δ*: 13.05 (br s, 1H), 8.45 (d, *J* = 2.1 Hz, 1H), 8.29 (dd, *J* = 8.7 Hz, *J* = 2.1 Hz, 1H), 8.20 (s, 1H), 6.90 (d, *J* = 8.7 Hz, 1H), 4.29 (br s, 4H), 3.76 (t, *J* = 4.8 Hz, 4H); ^13^C (75 MHz, DMSO-d6) *δ*: 154.5, 152.9, 139.3, 139.1, 132.2, 131.2, 130.6, 128.9, 127.5, 118.2, 66.2, 45.2.

#### 3.2.19. 4-(2-(4-Chloro-3-(trifluoromethyl)phenyl)-9H-purin-6-yl)morpholine **3s**

Method A: Compound **2b** (0.27 g, 1.38 mmol) in EtOH, aldehyde **4o** (1.1 eq.), and Et_3_N (10 eq.) was kept at 22 °C for 7 days and then at 40 °C for 5 days. The product **3u** was isolated as an off-white solid (0.28 g, 0.76 mmol, 55%); m.p. = 248–250 °C (Found: C, 50.00; H, 3.53; N, 18.28. C_16_H_13_ClF_3_N_5_O requires C, 50.00; H, 3.39; N, 18.25); IR (Nujol mull) νmax: 3191, 1587, 1575 cm^−1^; ^1^H (300 MHz, DMSO-d6) *δ*: 13.24 (s, 1H, NH), 8.72 (d, *J* = 2.1 Hz, 1H), 8.60 (dd, *J* = 8.4 Hz, *J* = 2.1 Hz, 1H), 8.21 (s, 1H), 7.82 (d, *J* = 8.4 Hz, 1H), 4.30 (br s, 4H), 3.76 (t, *J* = 4.5 Hz, 4H); ^13^C (75 MHz, DMSO-d6) *δ*: 156.3, 152.9, 152.4, 139.3, 137.9, 132.6, 131.8, 131.6 (q, *J* = 1Hz), 126.6 (q, *J* = 1Hz), 126.6 (q, *J* = 31 Hz), 126.0 (q, *J* = 6 Hz), 122.9 (q, *J* = 271 Hz), 118.3, 66.2, 45.1. 

#### 3.2.20. 4-(2-(2,5-Dichlorophenyl)-9H-purin-6-yl)morpholine **3t**

Method A: Compound **2b** (0.28 g, 1.45 mmol) in EtOH, aldehyde **4h** (1.1 eq.), and Et_3_N (10 eq.) was kept at 22 °C for 6 days and then at 40 °C for 6 days. The product **3r** was isolated as an off-white solid (0.21 g, 0.60 mmol, 41%); m.p. = 259–261 °C (Found: C, 51.26; H, 3.69; N, 19.98. C_15_H_13_Cl_2_N_5_O requires C, 51.45; H, 3.74; N, 20.00); IR (Nujol mull) νmax: 3090, 1602, 1586, 1561 cm^−1^; ^1^H (300 MHz, DMSO-d6) *δ*: 13.23 (br s, 1H, NH), 8.22 (s, 1H), 7.76 (d, *J* = 2.1 Hz, 1H), 7.57 (d, *J* = 8.7 Hz, 1H), 7.50 (dd, *J* = 8.7 Hz, *J* = 2.1 Hz, 1H), 4.24 (br s, 4H), 3.72 (t, *J* = 4.5 Hz, 4H); ^13^C (75 MHz, DMSO-d6) *δ*: 156.3, 152.7, 152.0, 140.2, 139.3, 131.9, 131.4, 131.0, 130.4, 129.6, 117.7, 66.2, 45.2.

#### 3.2.21. 5-Chloro-2-(6-morpholino-9H-purin-2-yl)phenol **3u**

Method B: Compound **2b** (0.18 g, 0.91 mmol) in EtOH, aldehyde **4k** (1.1 eq.), and TFA (1.0 eq.) was kept at 22 °C for 2 days. Then, the reaction continued in EtOH and Et_3_N (10 eq.) at 40 °C for 24 days. The product **3s** was isolated as an off-white solid (0.08 g, 0.24 mmol, 26%); m.p. > 300 °C. (Found: C, 54.33; H, 4.23; N, 21.31. C_15_H_14_ClN_5_O_2_ requires C, 54.31; H, 4.25; N, 21.11); ^1^H (400 MHz, DMSO-d6) *δ*: 13.76 (s, 1H), 13.32 (br s, 1H), 8.27 (d, *J* = 2.8 Hz, 1H), 8.23 (s, 1H), 7.34 (dd, *J* = 8.8 Hz, *J* = 2.8 Hz, 1H), 6.94 (d, *J* = 8.8 Hz, 1H), 4.27 (br s, 4H), 3.78 (t, *J* = 4.8 Hz, 4H); ^13^C (100 MHz, DMSO-d6) *δ*: 158.2, 156.2, 152.5, 149.6, 139.3, 131.4, 127.7, 122.2, 120.7, 119.2, 117.9, 66.1, 45.4.

#### 3.2.22. 4-(2-(2-Fluoro-5-methoxyphenyl)-9H-purin-6-yl)morpholine **3v**

Method A: Compound **2b** (0.35 g, 1.80 mmol) in EtOH, aldehyde **4l** (1.1 eq.), and Et_3_N (10 eq.) was kept at 20 °C for 19 days and then at 40 °C for 7 days. The product **3v** was isolated as an off-white solid (0.17 g, 0.52 mmol, 29%); m.p. = 261–263 °C (Found: C, 57.99; H, 4.89; N, 20.98. C_16_H_16_FN_5_O_2_ requires C, 58.35; H, 4.90; N, 21.27); IR (Nujol mull) νmax: 1579, 1500 cm^−1^; ^1^H (300 MHz, DMSO-d6) *δ*: 13.17 (s, 1H), 8.18 (s, 1H), 7.50 (dd, *J* = 6.6 Hz, *J* = 3.6 Hz, 1H), 7.17 (dd, *J* = 10.5 Hz, *J* = 9.0 Hz, 1H), 7.02 (dt, *J* = 3.6 Hz, *J* = 3.3 Hz, 1H), 4.24 (br s, 4H), 3.77 (s, 3H), 3.72 (m, 4H); ^13^C (75 MHz, DMSO-d6) *δ*: 154.7 (d, *J* = 244 Hz), 155.2 (d, *J* = 5 Hz), 155.0 (d, *J* = 2 Hz), 152.9, 152.3, 139.2, 127.8 (d, *J* = 12 Hz), 117.7, 117.4 (d, *J* = 25 Hz), 116.0 (d, *J* = 8 Hz), 115.7 (d, *J* = 2 Hz), 66.2, 55.6, 45.1.

#### 3.2.23. 4-(2-(2,5-Difluorophenyl)-9H-purin-6-yl)morpholine **3x**

Method A: Compound **2b** (0.31 g, 1.59 mmol) in EtOH, aldehyde **4m** (1.1 eq.), and Et_3_N (10 eq.) was kept at 35 °C for 1 month. The product **3x** was isolated as an off-white solid (0.28 g, 0.88 mmol, 56%); m.p. = 260–262 °C (Found: C, 56.78; H, 4.00; N, 21.89. C_15_H_13_F_2_N_5_O requires C, 56.78; H, 4.13; N, 22.07); IR (Nujol mull) νmax: 1581, 1500 cm^−1^; ^1^H (300 MHz, DMSO-d6) *δ*: 13.22 (s, 1H), 8.19 (s, 1H), 7.75–7.80 (m, 1H), 7.29–7.33 (m, 2H), 4.24 (br s, 4H), 3.73 (br s, 4H); ^13^C (75 MHz, DMSO-d6) *δ*: 157.8 (dd, *J* = 240 Hz, *J* = 4 Hz), 156.6 (dd, *J* = 250 Hz, *J* = 4 Hz), 154.2 (d, *J* = 3 Hz), 152.9, 152.2, 139.3, 128.7 (dd, *J* = 11 Hz, *J* = 8 Hz), 118.4 (dd, *J* = 25 Hz, *J* = 9 Hz), 117.9, 117.4 (dd, *J* = 23 Hz, *J* = 11 Hz), 117.3 (dd, *J* = 23 Hz, *J* = 5 Hz), 66.2, 45.2. 

### 3.3. Radioligand Binding Assays

The %_inhib_ of specific radioligand binding at the receptors by the compounds was tested at the concentration of 10 µM at all adenosine receptors following the conditions described below. Competition binding curves at several receptors were made by testing six different concentrations (ranging from 10 nM to 100 µM) for all of the compounds showing an %_inhib_ ≥ 80%.

#### 3.3.1. Human A_1_ Receptor

Competition binding experiments of adenosine A_1_ receptor were made with membranes from CHO-A_1_ cells (Euroscreen, Brussels, Belgium). Membranes were defrosted and suspended in an incubation buffer with 10 mM of MgCl_2_, 100 mM of NaCl, 20 mM of Hepes, 2 UI/mL of adenosine deaminase, and pH = 7.4. Each well of a GF/C multiscreen plate (Millipore, Madrid, Spain), prepared in duplicate, contained 15 μg of protein, 2 nM of [^3^H]DPCPX, and the tested compound. Non-specific binding was calculated with 10 μM of (R)-PIA. The reaction mixture was incubated at room temperature (25 °C) for 60 min, after which samples were filtered and measured in a microplate beta scintillation counter (Microbeta Trilux, Perkin Elmer, Madrid, Spain).

#### 3.3.2. Human A_2A_ Receptor

Competition binding experiments of adenosine A_2A_ receptor were made in membranes from HeLa-A_2A_ cells, which were provided by Dr. Mengod (Departament de Neuroquimica, Institut d’Investi-gacions Biomediques de Barcelona, CSIC-IDIBAPS, Barcelona, Spain). Membranes were defrosted and suspended in an incubation buffer with 1 mM of EDTA, 50 mM of Tris-HCl, 10 mM of MgCl_2_, and 2 UI/mL of adenosine deaminase, with pH = 7.4. Each well of a GF/C multiscreen plate (Millipore, Madrid, Spain), prepared in duplicate, contained 3 nM of [^3^H]ZM241385, 10 µg of protein, and the tested compound. Nonspecific binding was calculated with 50 μM of NECA. The reaction mixture was incubated at room temperature (25 °C) for 30 min, after which samples were filtered and measured in a microplate beta scintillation counter (Microbeta Trilux, Perkin Elmer, Madrid, Spain).

#### 3.3.3. Human A_2B_ Receptor

Competition binding experiments of adenosine A_2B_ receptor were made in membranes from HEK-293-A_2B_ cells (Euroscreen, Brussels, Belgium). Membranes were defrosted and suspended in incubation buffer with 1 mM of EDTA, 10 mM of MgCl_2_, 0.1 mM of benzamidine, 50 mM of Tris-HCl, 10 µg/mL of bacitracine, and 2 UI/mL of adenosine deaminase, with pH = 6.5. Each reaction well, prepared in duplicate, contained 35 nM of [^3^H]DPCPX, 18 µg of protein, and the tested compound. Non-specific binding was calculated with 400 µM of NECA. The reaction mixture was incubated at room temperature (25 °C) for 30 min, after which samples were filtered through a multiscreen GF/C microplate and measured in a microplate beta scintillation counter (Microbeta Trilux, Perkin Elmer, Madrid, Spain).

#### 3.3.4. Human A_3_ Receptor

Competition binding experiments of adenosine A_3_ receptor were made in membranes from HeLa-A_3_ cells, which were provided by Dr. Mengod (Departament de Neuroquimica, Institut d’Investi-gacions Biomediques de Barcelona, CSIC-IDIBAPS, Barcelona, Spain). Membranes were defrosted and suspended in incubation buffer with 1 mM of EDTA, 5 mM of MgCl_2_, 50 mM of Tris-HCl, and 2 UI/mL of adenosine deaminase, with pH = 7.4. Each reaction well of a GF/B multiscreen plate (Millipore, Madrid, Spain), prepared in triplicate, contained 90 µg of protein, 30 nM of [^3^H]NECA, and the tested compound. Non-specific binding was determined with 100 µM of (R)-PIA. The reaction mixture was incubated at room temperature (25 °C) for 180 min, after which samples were filtered and measured in a microplate beta scintillation counter (Microbeta Trilux, Perkin Elmer, Madrid, Spain).

### 3.4. Functional Studies

#### 3.4.1. Human A_1_ Receptor

The agonist/antagonist behavior of tested compounds at A_1_ receptor was determined in CHO-A_1_ cells by measuring reversion induced by the tested compound of 10 µM of NECA-mediated inhibition of forkolin-stimulated cAMP production. Cells grown in 96-well plates with growth medium containing dialyzed fetal bovine serum were washed 2 times with F-12 nutrient mixture medium containing 25 mM of HEPES and 20 µM of the phosphodiesterase inhibitor rolipram, with pH = 7.4. After this time, the tested compounds were preincubated for 15 min in test medium at different concentrations (ranging from 0.1 nM to 10 µM). After this time, 10 µM of NECA and 3 µM of forskolin were added to each well. The incubation was continued for 15 min, and the reaction was stopped with a lysis buffer from the cAMP enzyme immunoassay kit (GE Healthcare, Chicago, IL, USA). Cell lysates were put in a plate with anti-IgG and anti-cAMP in the presence of cAMP–peroxidase during 60 min. Then, the wells were washed 4 times with the wash buffer from the kit, and TMB was added to each well and incubated for 60 min. The peroxidase reaction was stopped with 1 M of sulphuric acid, and the cAMP amount was determined from the optical density at 450 nm (Tecan M100 reader).

#### 3.4.2. Human A_3_ Receptor

The agonist/antagonist behavior of tested compounds at A_3_ receptor was determined in CHO–A3 cells, which were in-house generated at BioFarma Research Group (Department of Pharma-cology, University of Santiago de Compostela, Spain), by measuring reversion induced by the tested compound of 10 µM of NECA inhibition of forskolin-stimulated cAMP production. Cells grown in 96-well plates with growth medium containing dialyzed fetal bovine serum were washed 2 times with DMEM F-12 nutrient medium containing 25 mM of HEPES, 30 μM of rolipram, with pH = 7.4. After this time, the tested compounds were preincubated for 15 min in test medium at different concentrations (ranging from 0.1 nM to 10 µM). Finally, 10 μM of NECA and 10 μM of forskolin were added to each well. The incubation lasted 15 min, and cAMP was determined through an enzyme immunoassay (Perkin Elmer) for Human A_1_ receptor.

### 3.5. Data Analysis

Concentration response curves were fitted with Prism 10.1.2 (Graph Pad, San Diego, CA, USA). Radioligand binding p*K_i_* values were extrapolated from the equation

p*K_i_* = −log(IC_50_/(1 + ([L]/K_D_)))

where IC_50_ is the concentration that displaced specific radioligand binding at 50%, extrapolated from non-linear fitting, [L] is the concentration of radioligand employed, and K_D_ is the dissociation constant of the radioligand. In cAMP studies, K_B_ values were derived by employing the derivation of the Cheng-Prusoff equation, reported by Leffand Dougall in 1993 [37].

K_B_ = IC_50_/(2 + (A/(EC_50_)^n^)^1/n^ − 1);

where A is the concentration of the agonist employed to stimulate the receptor, EC_50_ is the potency of the agonist for stimulating the receptor, and n is the Hill slope of the antagonist’s concentration-response curve.

### 3.6. Docking Studies

The molecular docking calculations were carried out using the Molecular Operating Environment (MOE) software (version 2022.02, https://www.chemcomp.com, accessed on 25 April 2024) and the crystal structure of the A_1_ receptor in complex with the ligand DU1 (PDB accession code: 5UEN) [35]. The receptor was prepared by removing non-protein atoms and adding missing side chains. Engineered mutations were reverted back to the wild-type sequence based on the side chain rotamers determined through MOE. The protonation states of ionizable residues were set to the most probable at physiological pH. The binding site was defined based on the co-crystallized ligand, and default parameters in MOE were used in the docking calculations.

## 4. Conclusions

In summary, several 6-morpholino-purine derivatives with different substituents in C-2 and a methyl or a hydrogen at N-9 of the purine ring were synthesized and pharmacologically tested at human A_1_, A_2A_, A_2B_, and A_3_ adenosine receptor subtypes. Four of the twenty-three synthesized purines showed high affinity and selectivity for A_1_ receptors, and five showed high affinity and selectivity for A_3_ receptors. Three compounds (**3k**, **3q**, and **3u**) showed a dual affinity for A_1_/A_2A_, A_1_/A_3_, and A_1_/A_2B_ receptors, respectively. In addition, three compounds showed high affinity without selectivity for any specific adenosine receptor subtype (**3n**, **3o**, and **3x**). SAR studies showed that the substituent groups in C-2, C-6, and N-9 positions affect the potency and selectivity of the compounds for adenosine receptors. To generate highly potent antagonist ligands for adenosine receptors, a morpholine group and a hydrogen atom must be present in C-6 and N-9, respectively. Indeed, 9H-purines bind some 5–358 times more strongly than their corresponding 9-CH_3_-purines for the adenosine receptors. The substituted aryl group in C-2 is very important for governing the selectivity towards the adenosine receptor subtypes. Additionally, our results suggested that the A_3_ receptor subtype presents a larger hydrophobic pocket than that of the A_1_ receptor subtype, as A_3_ accommodates bigger substituent groups Y present at the *meta* position of substituent R^1^. In conclusion, several purine-based compounds are described herein, some with high affinity and antagonist selectivity for A_1_, A_3_, dual A_1_/A_2A_, A_1_/A_2B_, and A_1_/A_3_ receptors.

## Figures and Tables

**Table 1:** Binding affinities of compounds **3** at all human adenosine receptors expressed as %_inhib_ at 10 µM or p*K_i_*. Values are reported as mean ± SEM of three experiments with duplicate measurements.

	R	R^1^	A_1_	A_2A_	A_2B_	A_3_
**a**	Me	2-ClC_6_H_4_	36 ± 1%	11 ± 2%	22 ± 5%	14 ± 2%
**b**	Me	2-MeOC_6_H_4_	22 ± 1%	12 ± 5%	12 ± 2%	36 ± 5%
**c**	Me	2-HOC_6_H_4_	**6.51 ± 0.11**	**6.47 ± 0.05**	**5.70 ± 0.07**	6 ± 5%
**d**	Me	3-ClC_6_H_4_	**6.80 ± 0.07**	15 ± 4%	2 ± 2%	25 ± 4%
**e**	Me	3-HOC_6_H_4_	**6.16 ± 0.21**	37 ± 1%	34 ± 3%	52 ± 4%
**f**	Me	4-ClC_6_H_4_	37 ± 4%	42 ± 5%	15 ± 4%	31 ± 5%
**g**	Me	2,3-(HO)_2_C_6_H_3_	33 ± 0.3%	25 ± 2%	22 ± 1.5%	42 ± 3%
**h**	Me	2,5-Cl_2_C_6_H_3_	41 ± 2%	2 ± 3%	22 ± 3%	8 ± 1%
**i**	Me	2,5-(HO)_2_C_6_H_3_	**6.36 ± 0.15**	**6.25 ± 0.12**	**6.32 ± 0.01**	53 ± 5%
**j**	Me	2-HO-5-MeOC_6_H_3_	**6.28 ± 0.17**	**5.91 ± 0.11**	**5.78 ± 0.03**	19 ± 11%
**k**	Me	2-HO-5-ClC_6_H_3_	**6.91 ± 0.08**	**6.15 ± 0.03**	55 ± 1%	53 ± 4%
**l**	Me	2-F-5-MeOC_6_H_3_	61 ± 4%	42 ± 1%	27 ± 2%	**5.28 ± 0.11**
**m**	Me	2,5-F_2_C_6_H_3_	**6.28 ± 0.13**	37 ± 2%	30 ± 4%	25 ± 3%
**n**	H	2-HOC_6_H_4_	**7.19 ± 0.10**	**6.97 ± 0.07**	**6.86 ± 0.10**	**6.59 ± 0.06**
**o**	H	3-ClC_6_H_4_	**7.75 ± 0.14**	**7.33 ± 0.07**	**7.12 ± 0.13**	**7.40 ± 0.14**
**p**	H	3-HOC_6_H_4_	**7.49 ± 0.06**	**6.26 ± 0.12**	60 ± 6%	**6.33 ± 0.08**
**q**	H	4-ClC_6_H_4_	**7.02 ± 0.07**	56 ± 3%	50 ± 4%	**7.47 ± 0.05**
**r**	H	3,4-Cl_2_C_6_H_3_	29 ± 1%	22 ± 5%	35 ± 3%	**7.15 ± 0.08**
**s**	H	3-CF_3_-4-ClC_6_H_3_	43 ± 5%	13 ± 5%	8 ± 5%	**6.02 ± 0.04**
**t**	H	2,5-Cl_2_C_6_H_3_	**5.97 ± 0.11**	44 ± 2%	26 ± 1%	**7.16 ± 0.17**
**u**	H	2-HO-5-ClC_6_H_3_	**7.05 ± 0.09**	35 ± 1%	**6.21 ± 0.08**	37 ± 1%
**v**	H	2-F-5-MeOC_6_H_3_	38 ± 2%	45 ± 4%	**6.40 ± 0.16**	**7.83 ± 0.16**
**x**	H	2,5-F_2_C_6_H_3_	**8.23 ± 0.06**	**7.15 ± 0.05**	**7.44 ± 0.11**	**7.63 ± 0.12**

**Table 2:** Antagonist potency (p*K*_B_) of the most potent compounds at human A_1_ and A_3_ adenosine receptors in cAMP tests. Values are reported as mean ± SEM of three experiments with duplicate measurements.

Compounds	Potency (p*K_B_*)	
	A_1_	A_3_
**3v**	N.D.	8.24 ± 0.11
**3x**	8.25 ± 0.16	N.D.

N.D.—Not determined.

## Data Availability

Data are contained within the article and Supplementary Materials.

## Supplementary Materials

The following supporting information can be downloaded at: https://www.mdpi.com/article/10.3390/molecules29112543/s1, ^1^H and ^13^C NMR spectra of representative synthesized compounds.
