# Supplementary material for: 2-Aryladenine Derivatives as a Potent Scaffold for Adenosine Receptor Antagonists: The 6-Morpholino Derivatives
Source: Molecules. 2024 May 28;29(11):2543. doi: 10.3390/molecules29112543 (PMC11173536; doi:10.3390/molecules29112543)
Supplement: Supplementary file 1 [file molecules-29-02543-s001.zip › molecules-2889107-supplementary.pdf]

2-Aryladenine Derivatives as a Potent Scaffold for Adenosine Receptor Antagonists – The 6-Morpholine Derivatives

Filipe Areias [1,2,3,4,†], Carla Correia [1,†], Ashly Rocha [1], Sofia Teixeira [1], Marián Castro [2,4], Jose Brea [2,4], Hubain Hu [5], Jens Carlsson [5], Maria I. Loza [2,4], M. Fernanda Proença [1], and M. Alice Carvalho [1,*]

[1] Centre of Chemistry of University of Minho (CQUM), Campus de Gualtar, Universidade do Minho, 4710-057 Braga, Portugal;
[2] Center for Research in Molecular Medicine and Chronic Diseases (CiMUS), Universidade de Santiago de Compostela, Avda de Barcelona, E-15782 Santiago de Compostela, Spain;
[3] School of Chemical Sciences & Engineering, Yachay Tech University, Yachay City of Knowledge, 100119 Urcuqui, Ecuador;
[4] Instituto de Investigación Sanitaria de Santiago de Compostela (IDIS), Travesía da Choupana s/n, E-15706 Santiago de Compostela, Spain;
[5] Science for Life Laboratory, Department of Cell and Molecular Biology, Uppsala University, SE-75124 Uppsala, Sweden;

*Correspondence: mac@quimica.uminho.pt (M.A.C.)
† These authors contributed equally to this work.

Table of contents

^{1}H and ^{13}C NMR spectra of representative synthesized compounds S2

Compound 3d

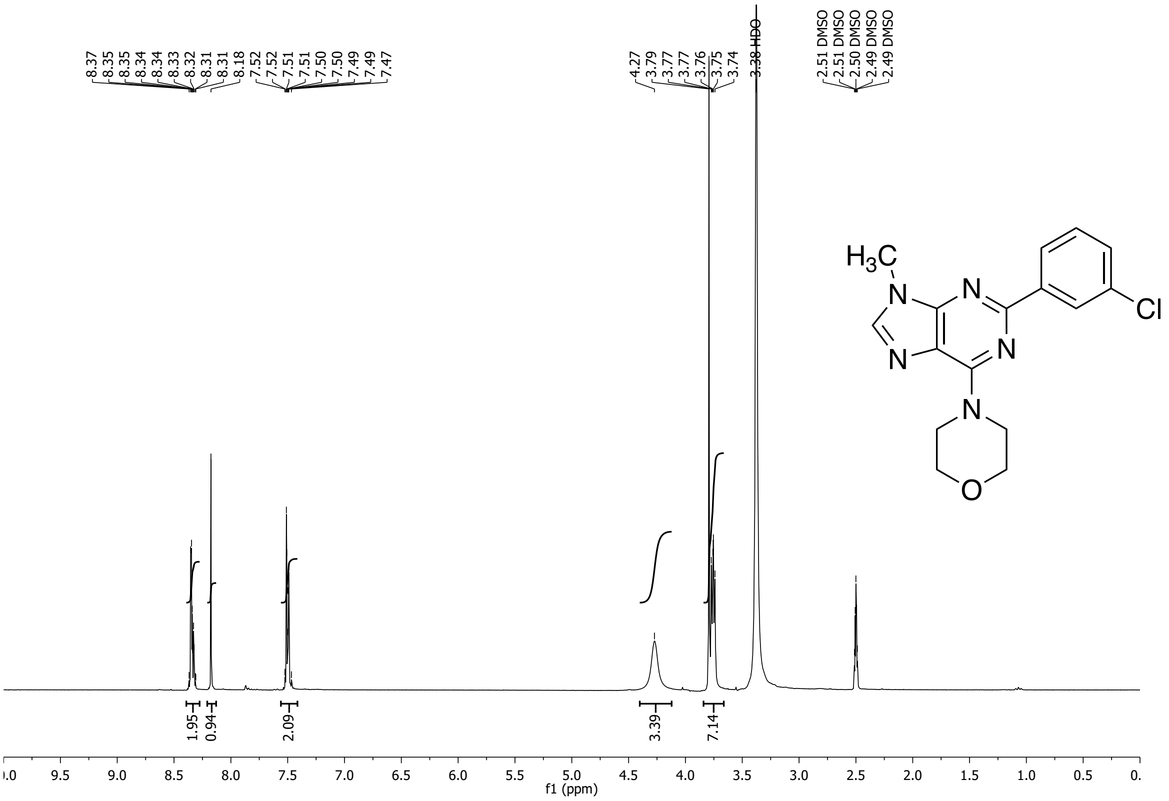

^1H NMR (300 MHz, DMSO-d6)

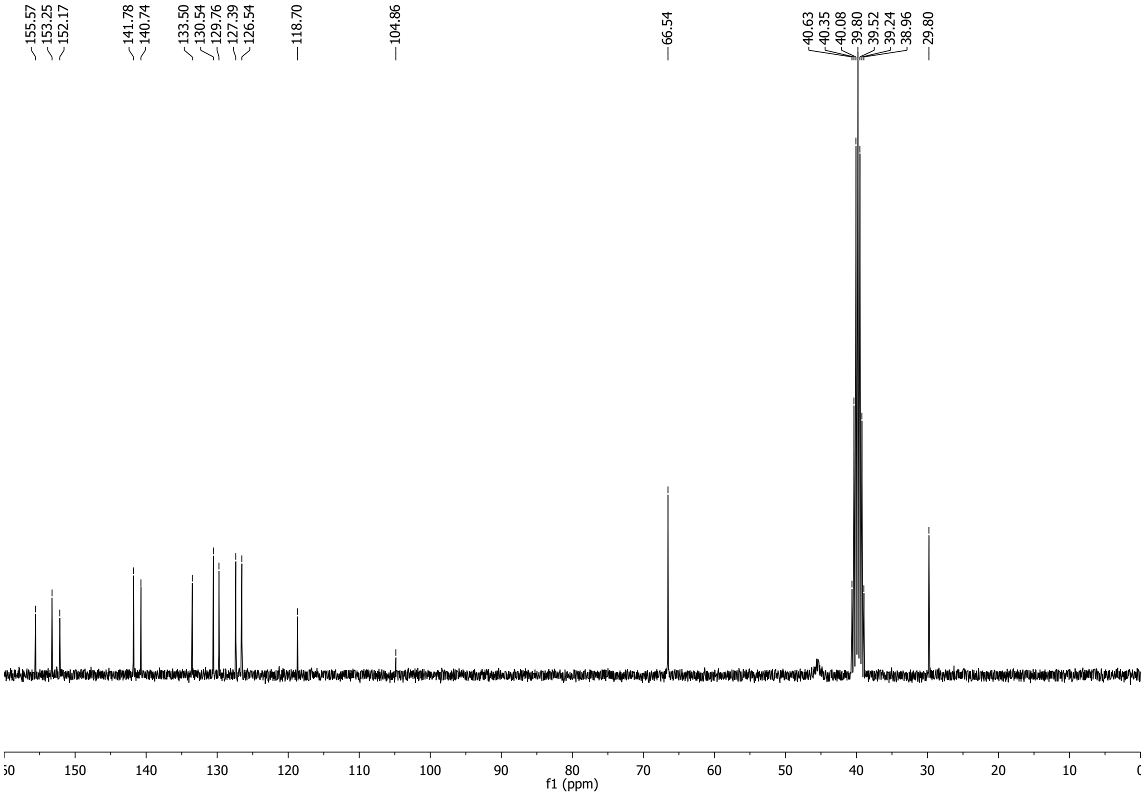

^{13}C NMR (75 MHz, DMSO-d6)

Compound 3e

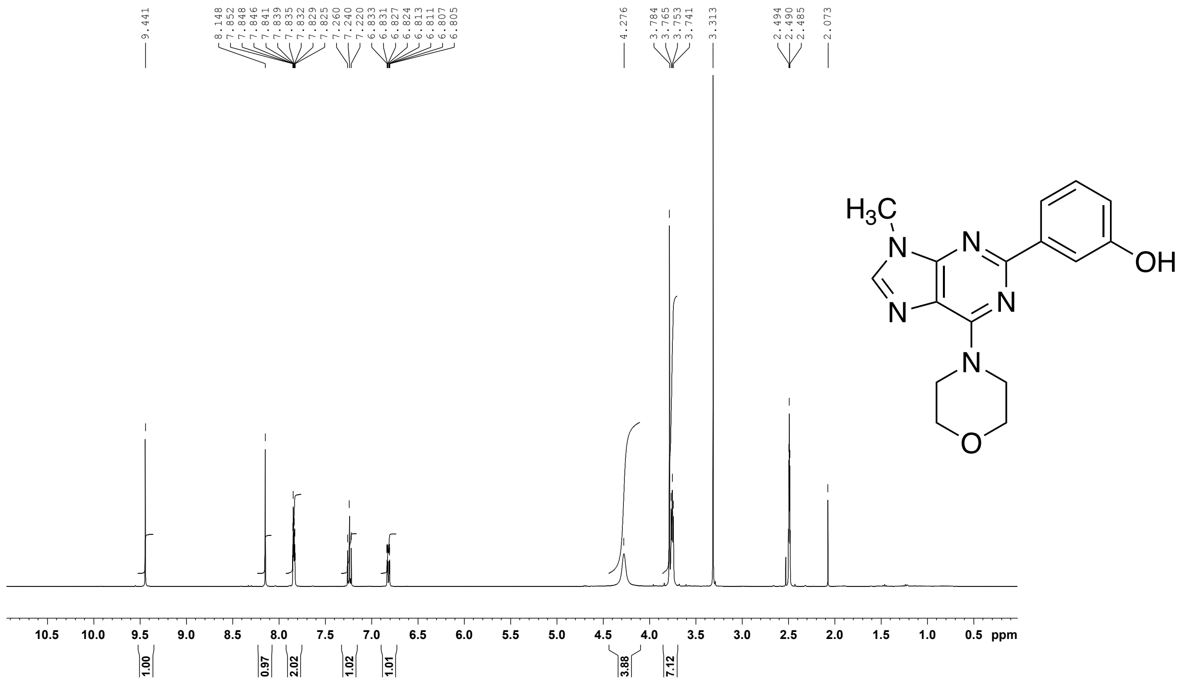

^{1}H NMR (300 MHz, DMSO-d6)

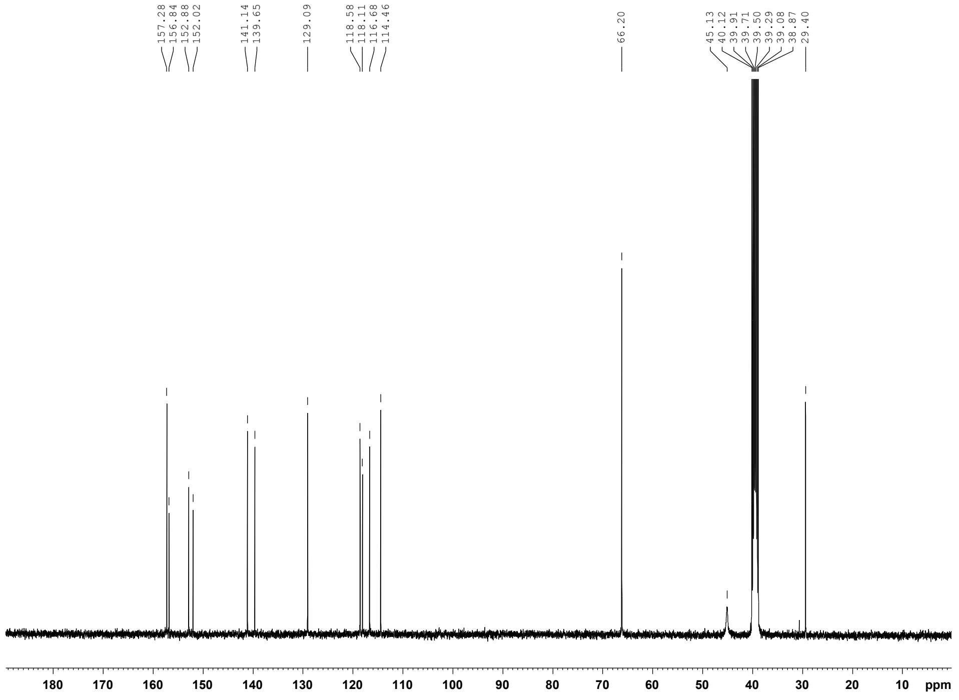

^{13}C NMR (75 MHz, DMSO-d6)

Compound 3g

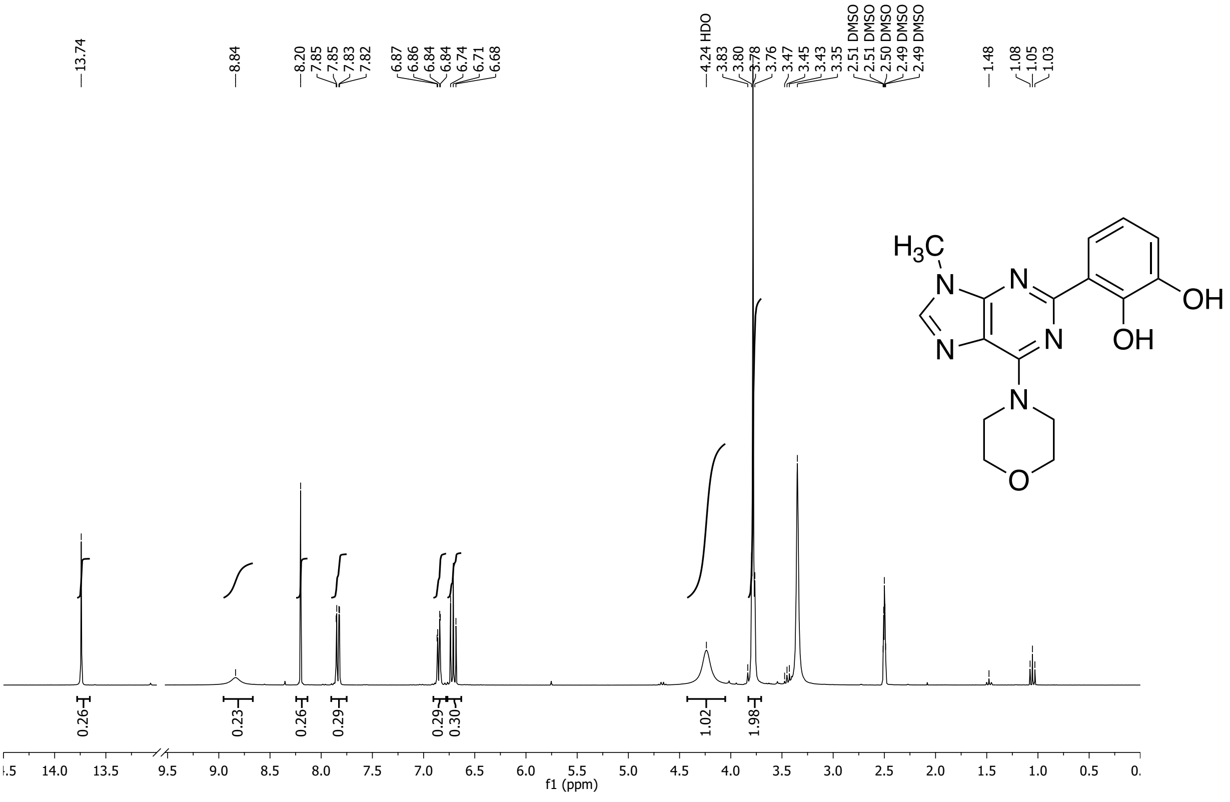

^{1}H NMR (300 MHz, DMSO-d6)

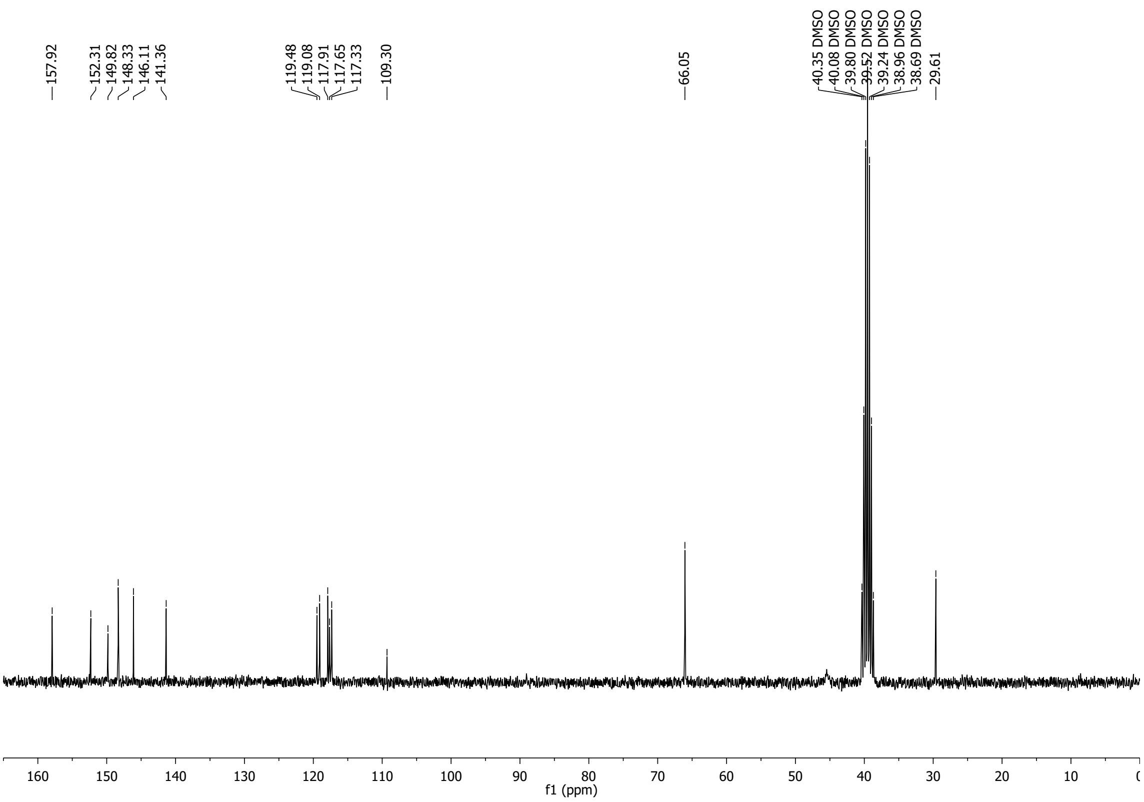

^{13}C NMR (75 MHz, DMSO-d6)

Compound 3h

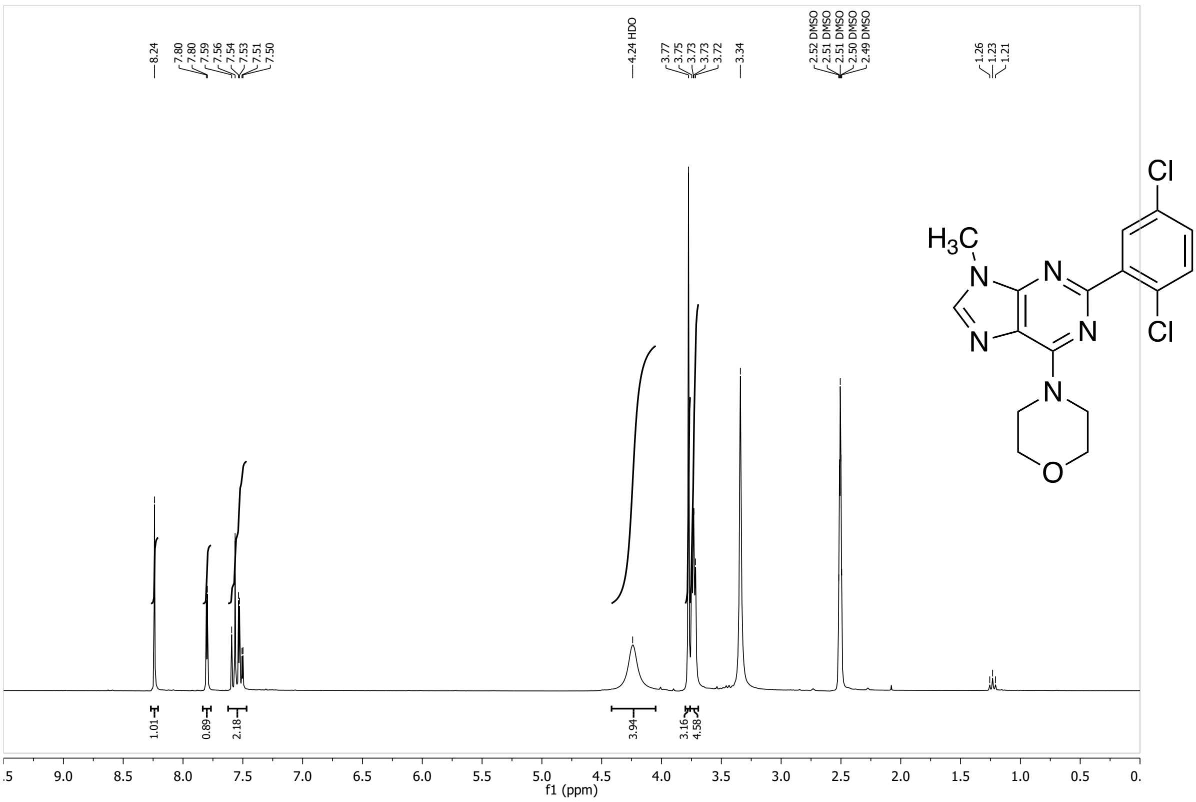

^{1}H NMR (300 MHz, DMSO-d6)

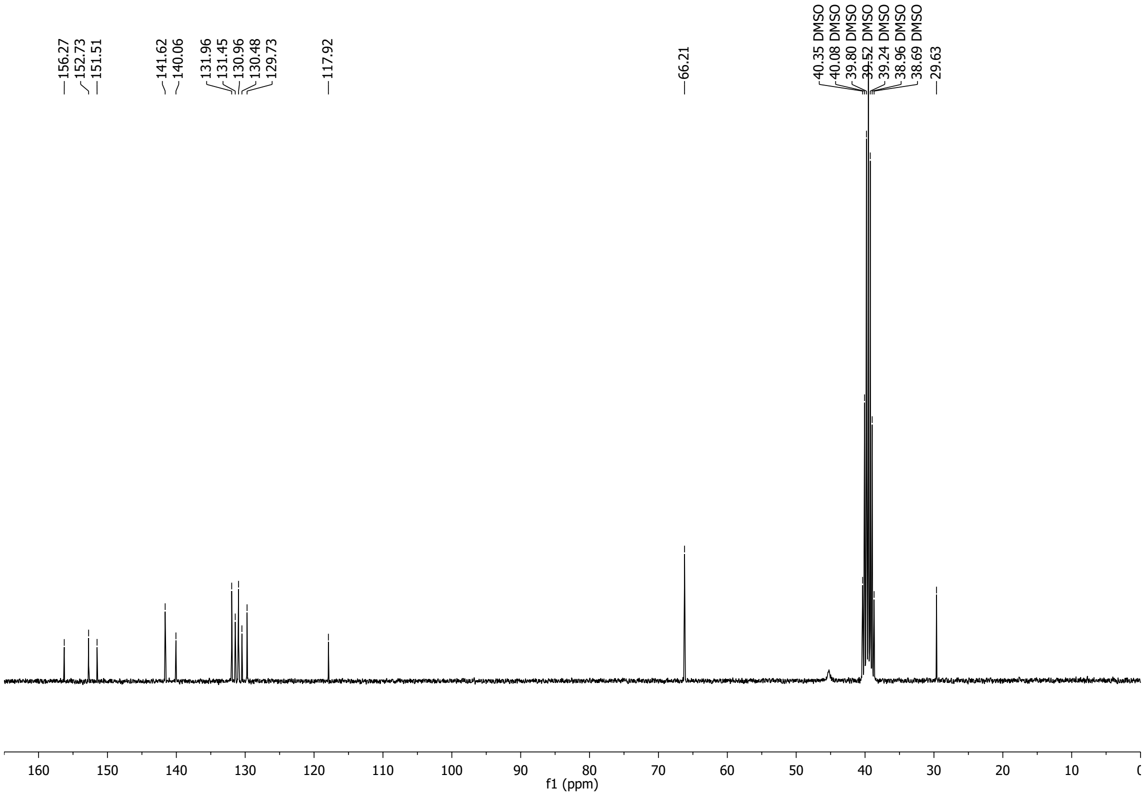

^{13}C NMR (75 MHz, DMSO-d6)

Compound 3i

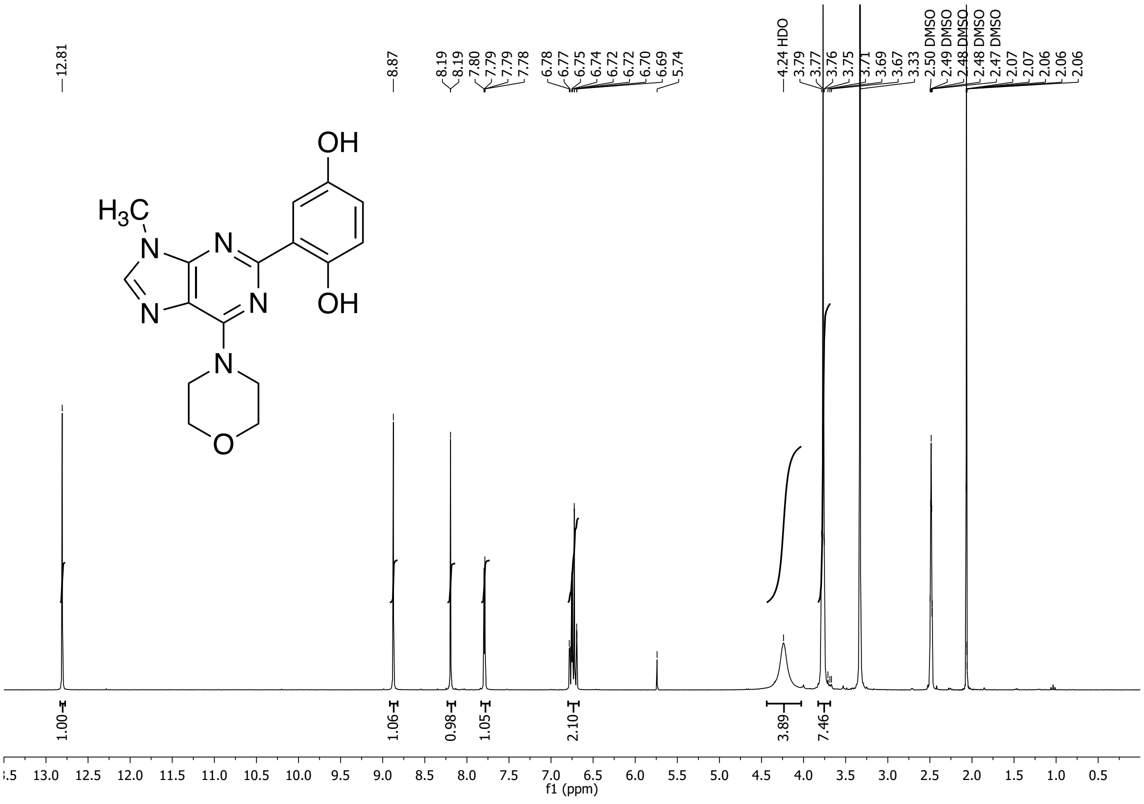

^{1}H NMR (300 MHz, DMSO-d6)

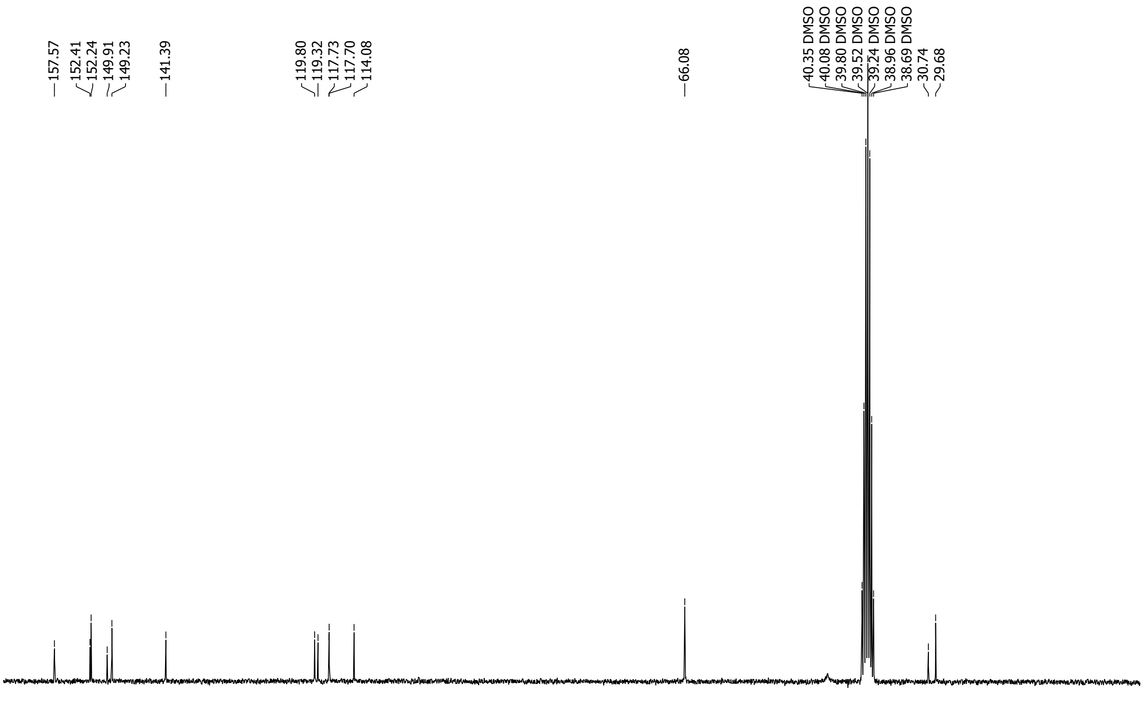

^{13}C NMR (75 MHz, DMSO-d6)

Compound 3k

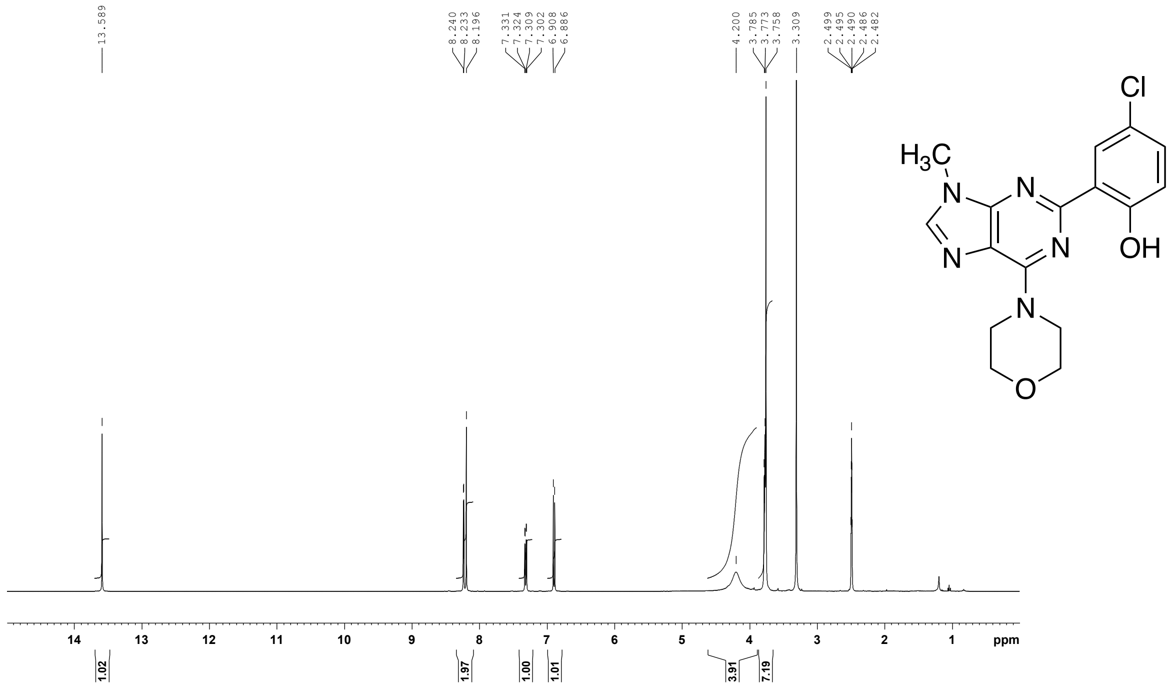

^{1}H NMR (300 MHz, DMSO-d6)

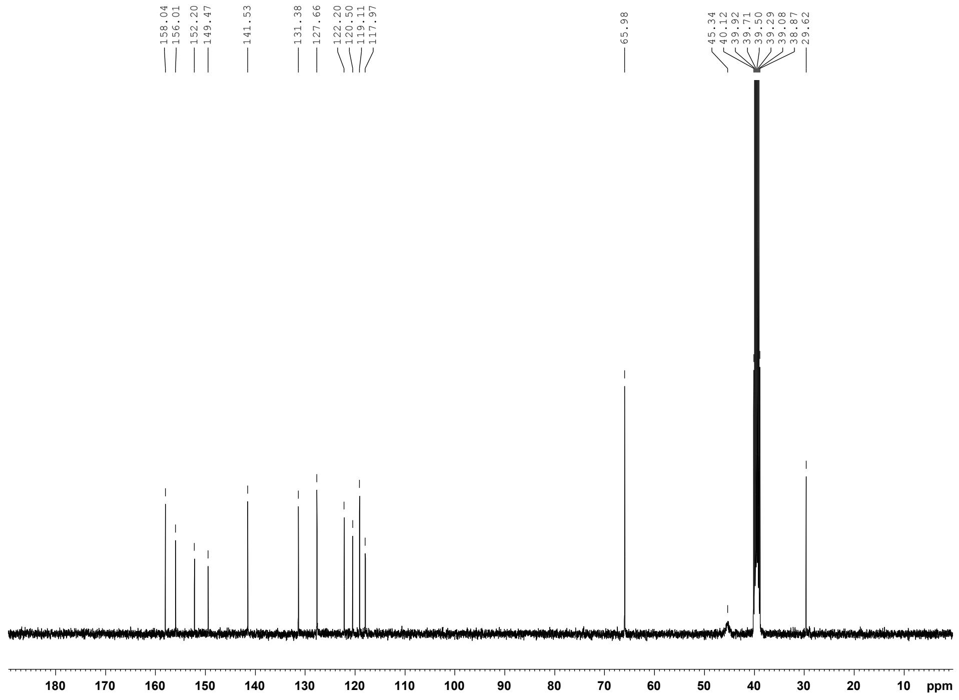

^{13}C NMR (75 MHz, DMSO-d6)

Compound 3l

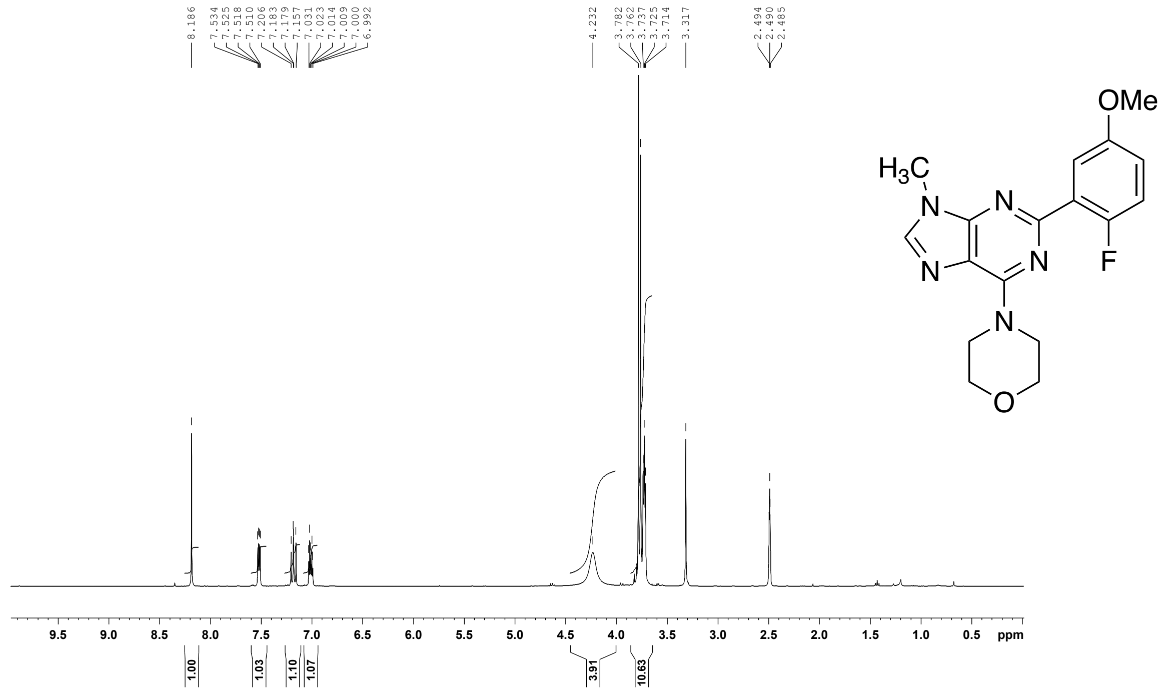

^{1}H NMR (300 MHz, DMSO-d6)

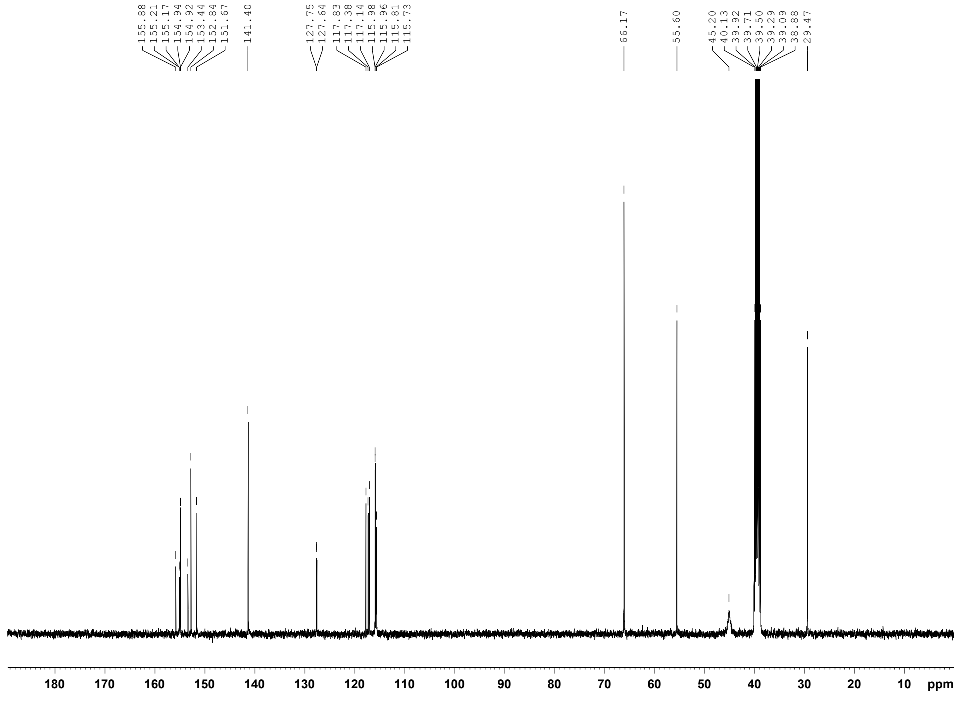

^{13}C NMR (75 MHz, DMSO-d6)

Compound 3m

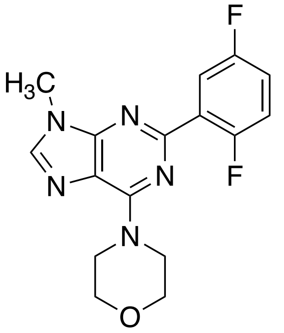

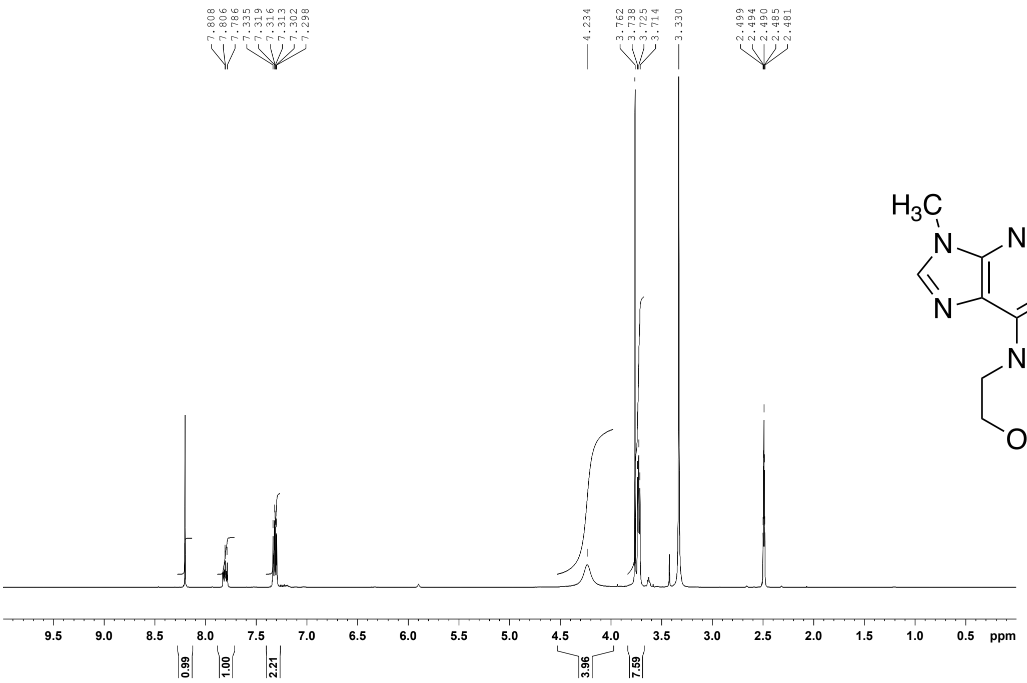

^{1}H NMR (400 MHz, DMSO-d6)

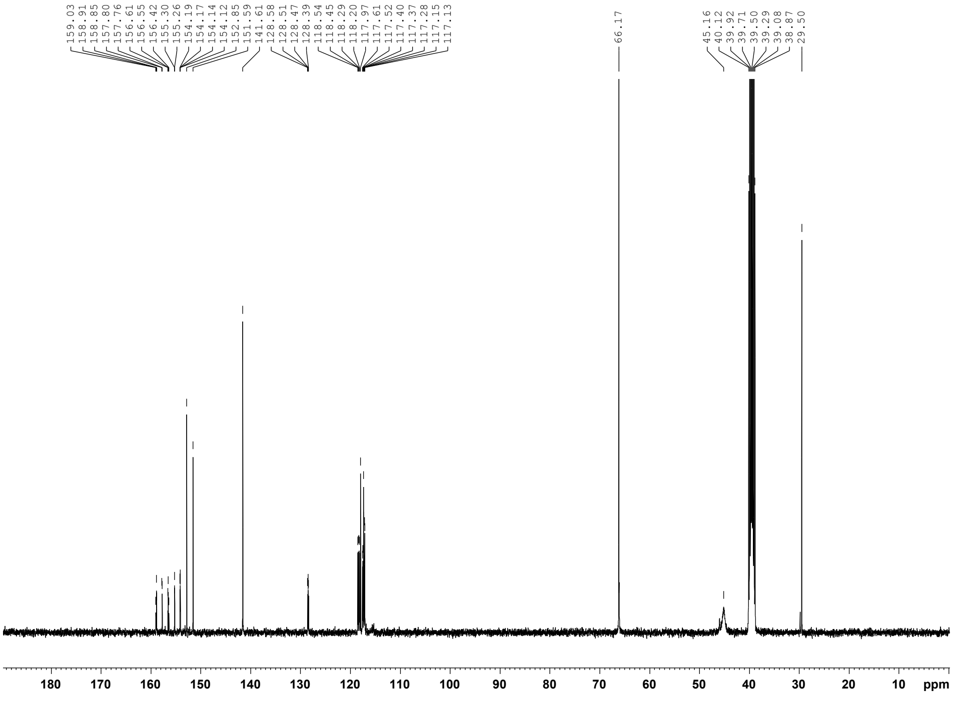

^{13}C NMR (100 MHz, DMSO-d6)

Compound 3n

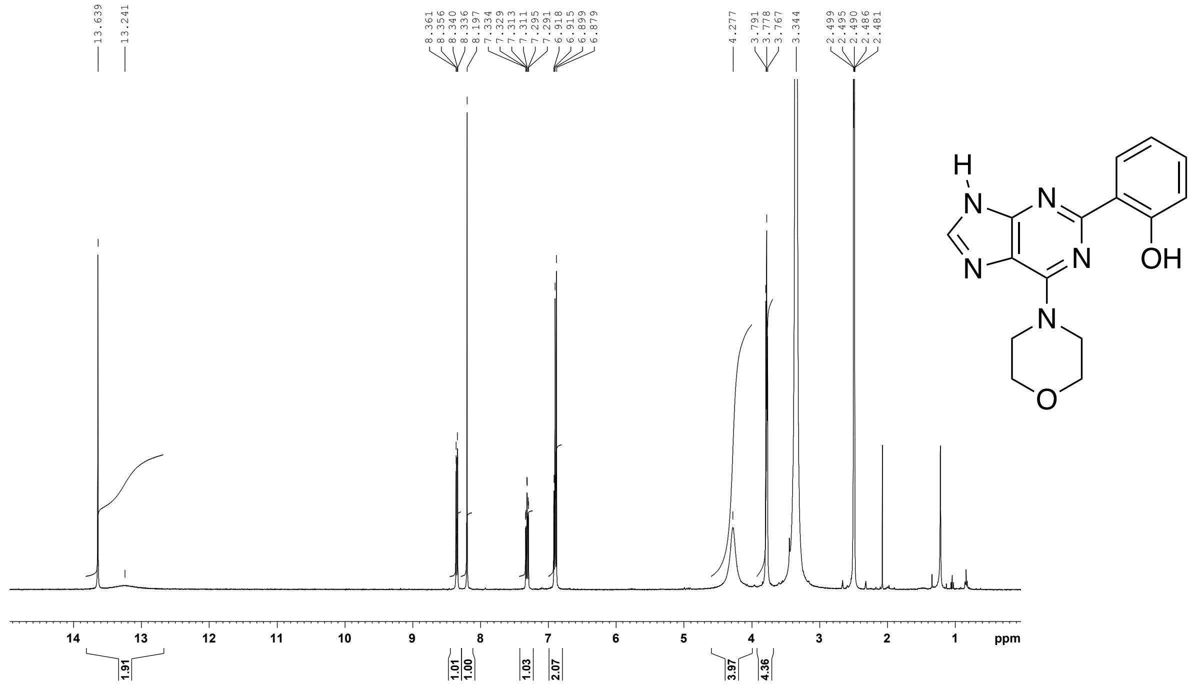

^{1}H NMR (400 MHz, DMSO-d6)

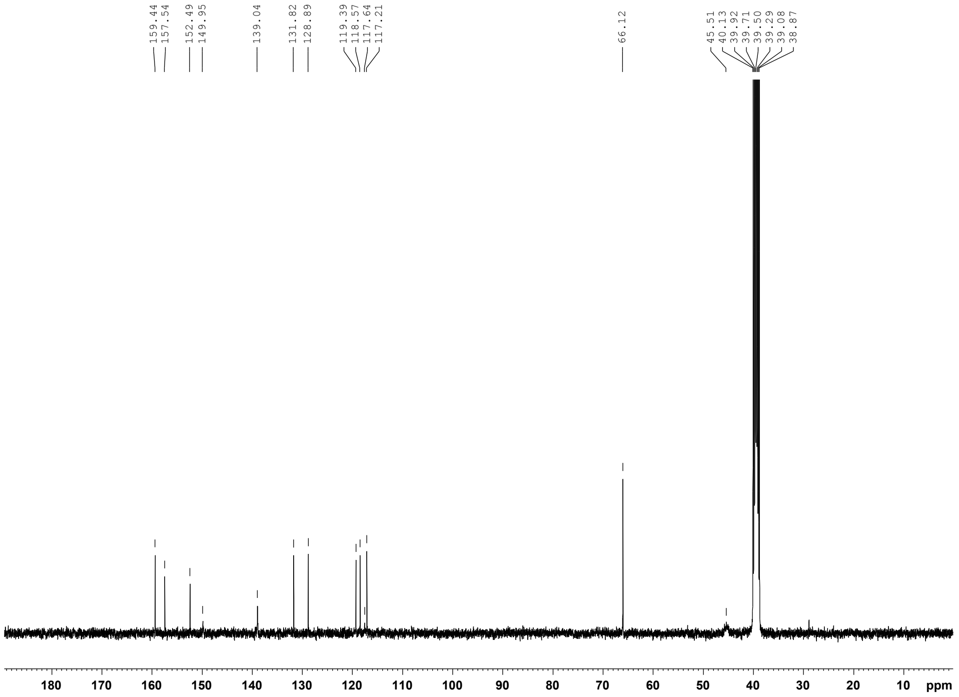

^{13}C NMR (100 MHz, DMSO-d6)

Compound 3o

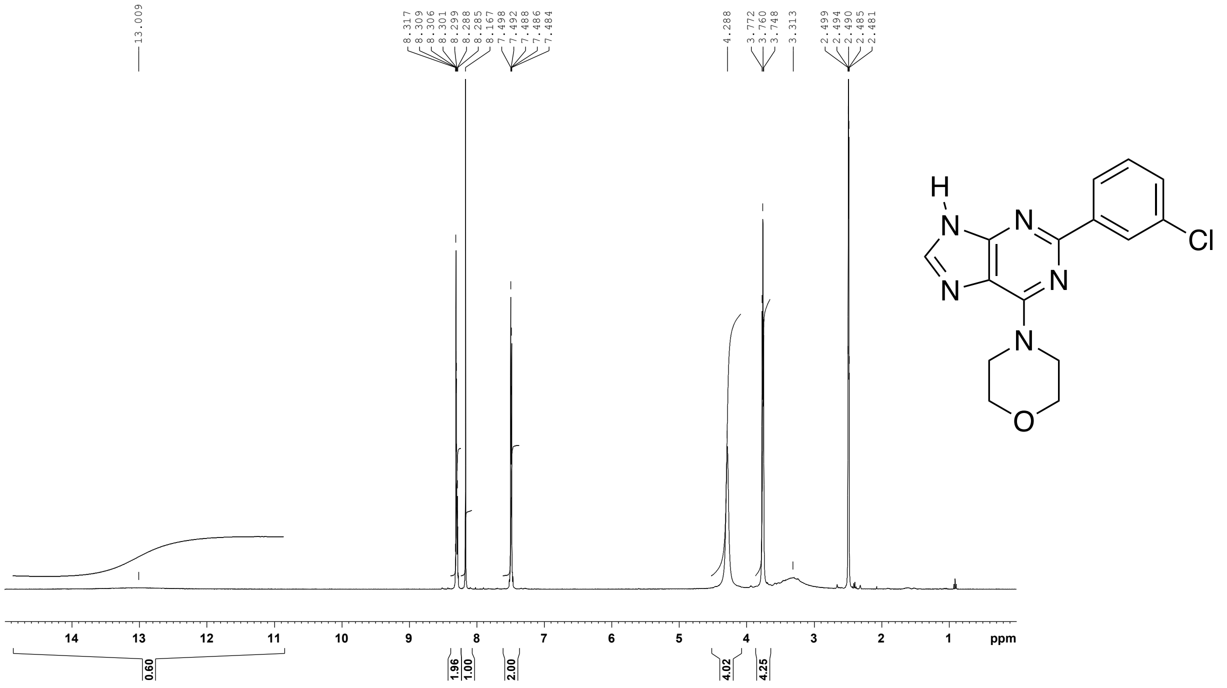

^{1}H NMR (300 MHz, DMSO-d6)

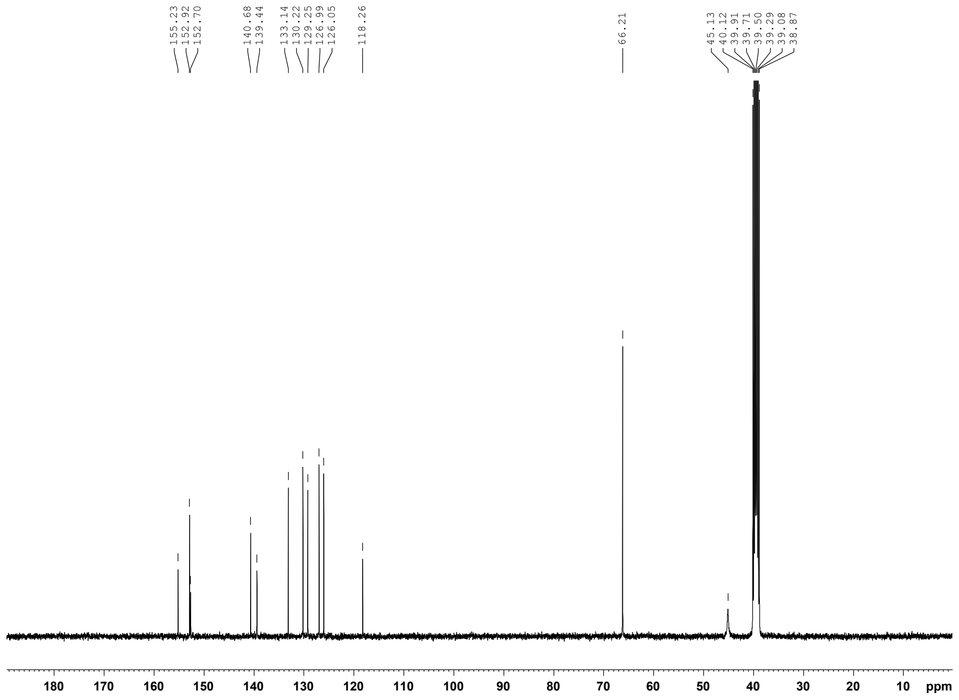

^{13}C NMR (75 MHz, DMSO-d6)

Compound 3p

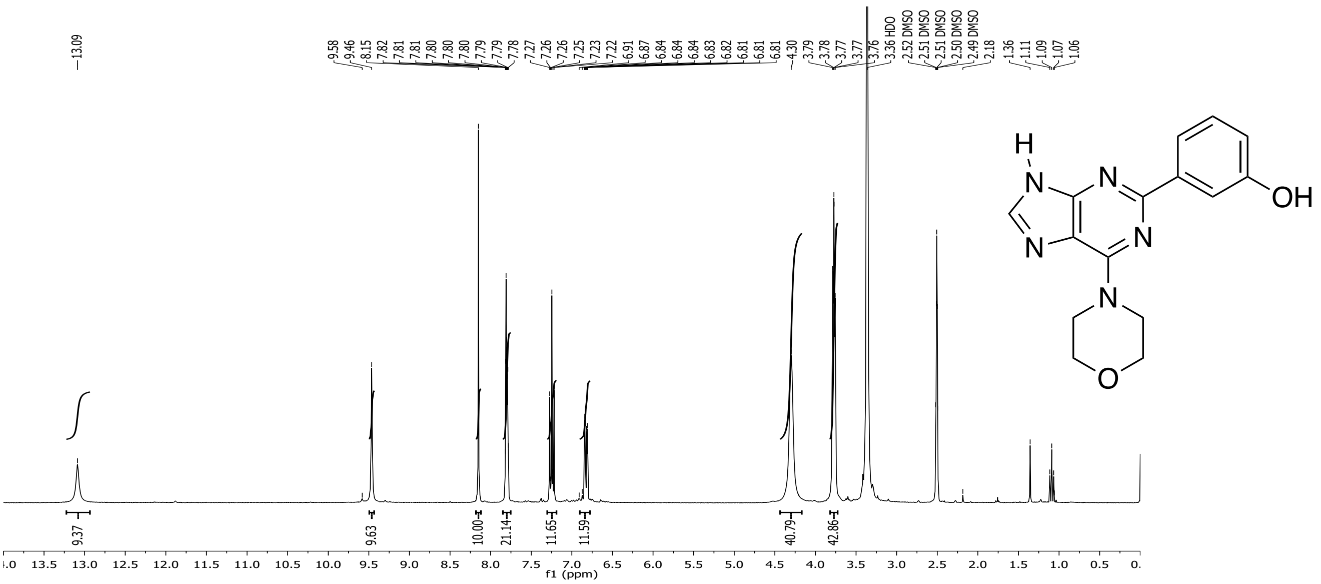

^{1}H NMR (300 MHz, DMSO-d6)

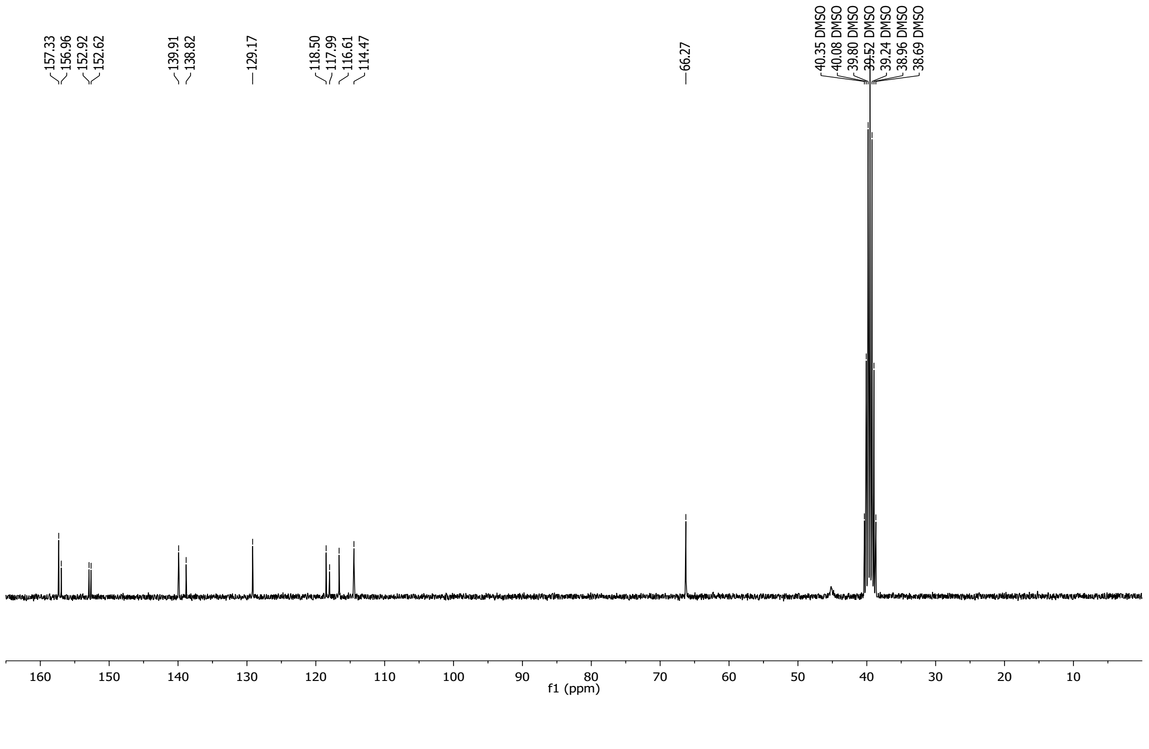

^{13}C NMR (75 MHz, DMSO-d6)

Compound 3r

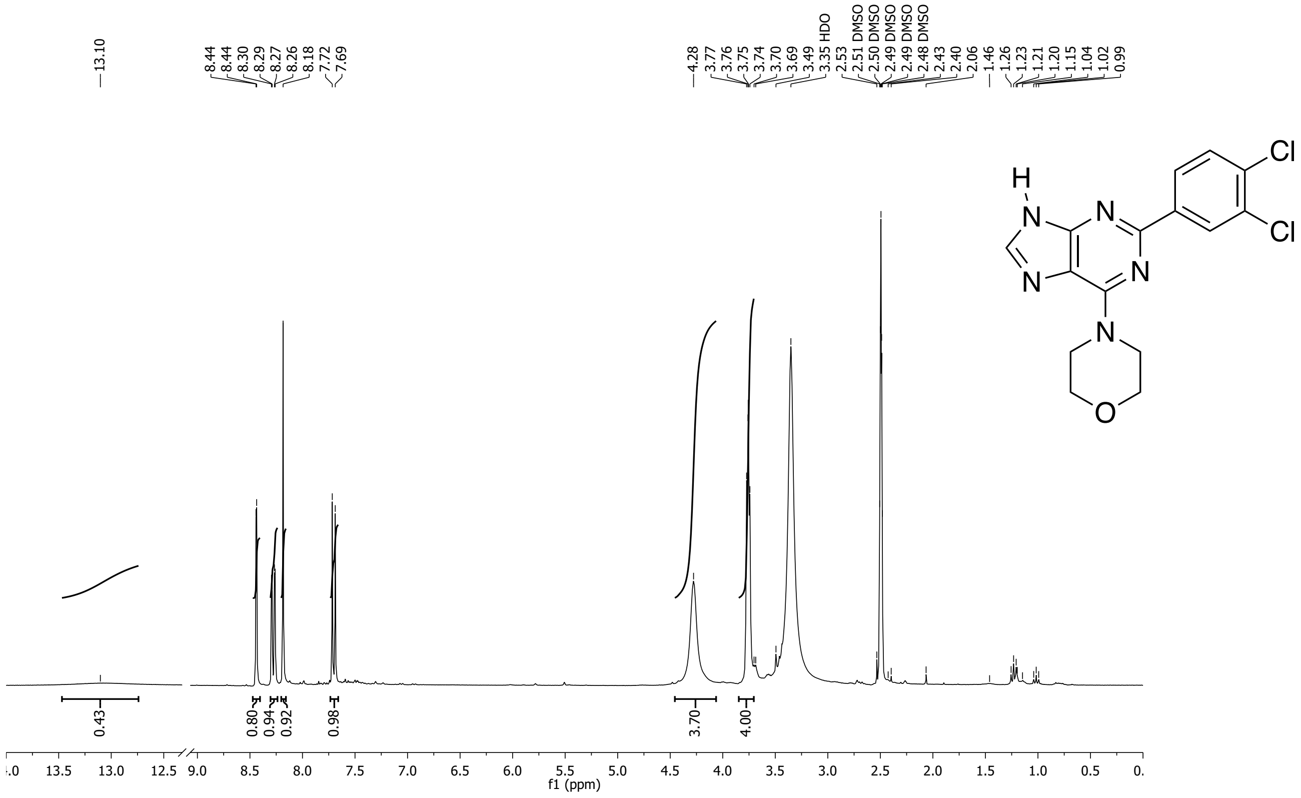

^{1}H NMR (300 MHz, DMSO-d6)

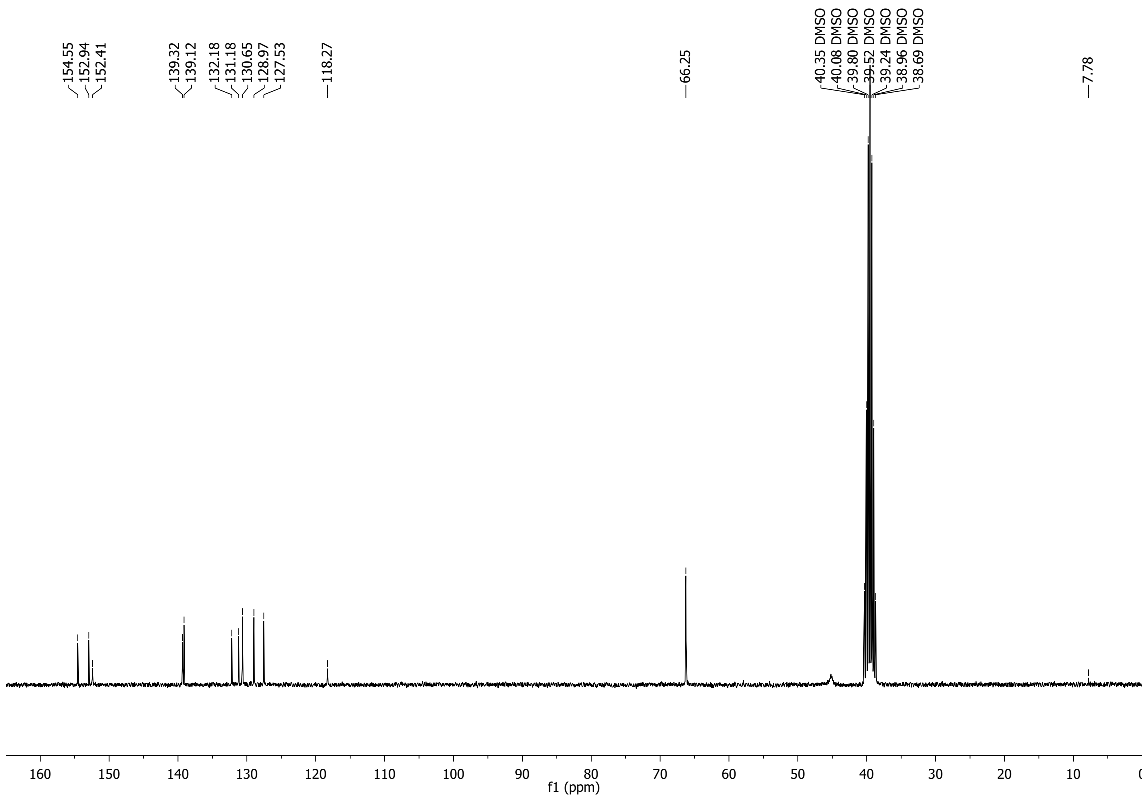

^{13}C NMR (75 MHz, DMSO-d6)

Compound 3s

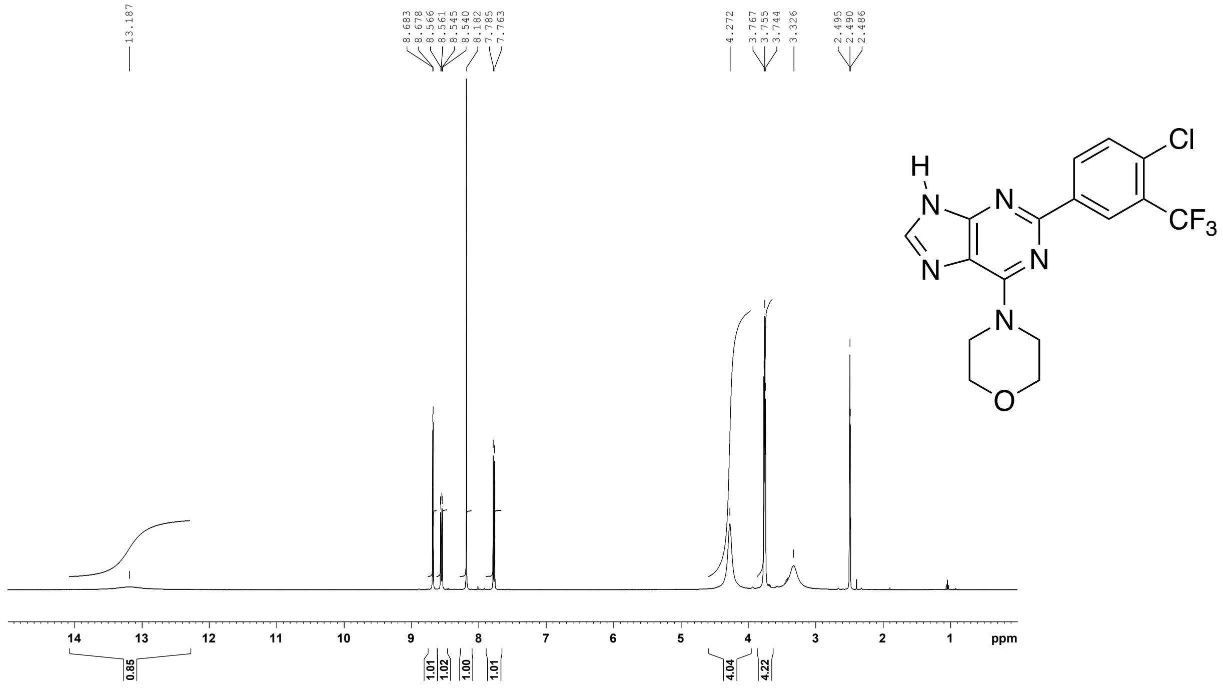

^{1}H NMR (300 MHz, DMSO-d6)

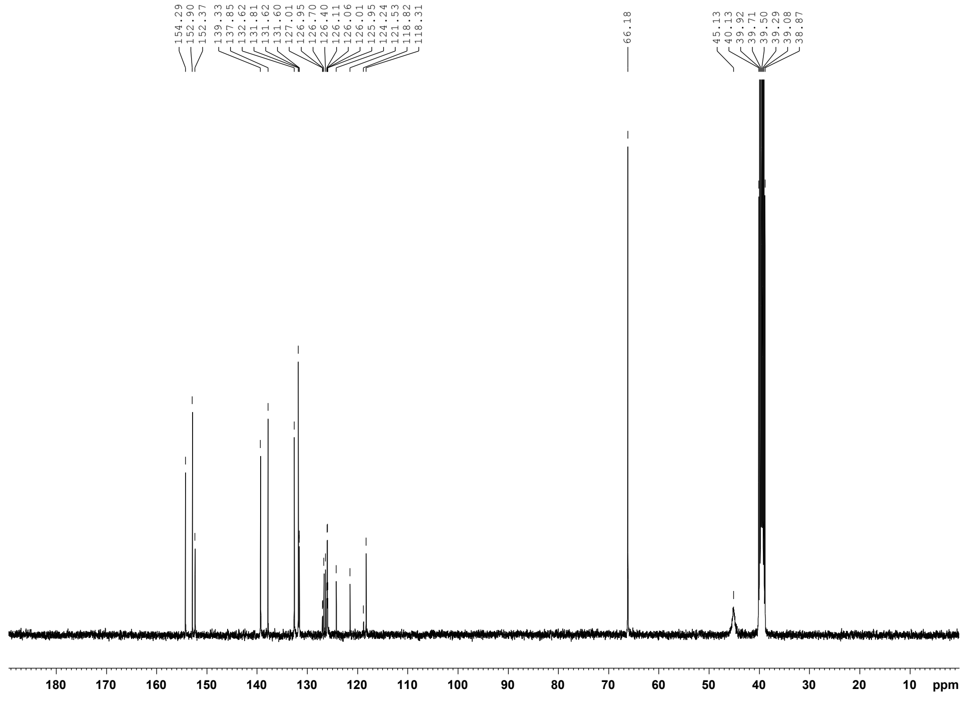

^{13}C NMR (75 MHz, DMSO-d6)

Compound 3t

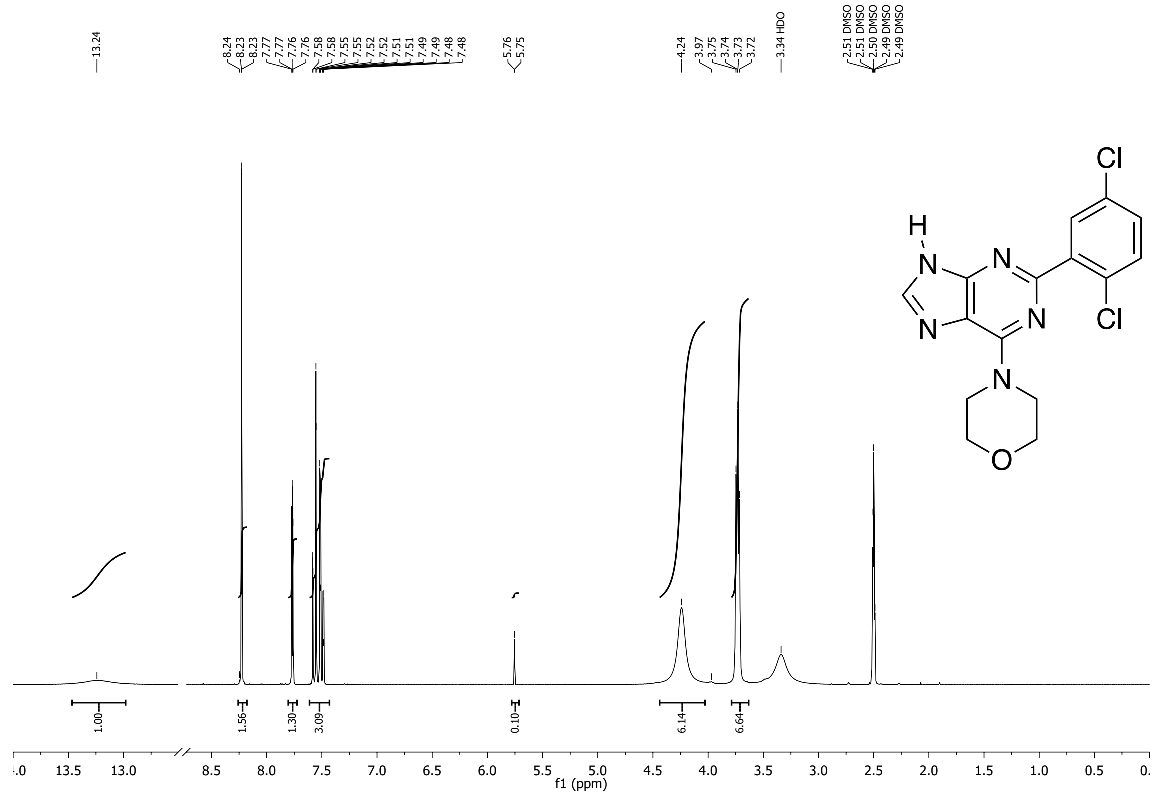

^{1}H NMR (300 MHz, DMSO-d6)

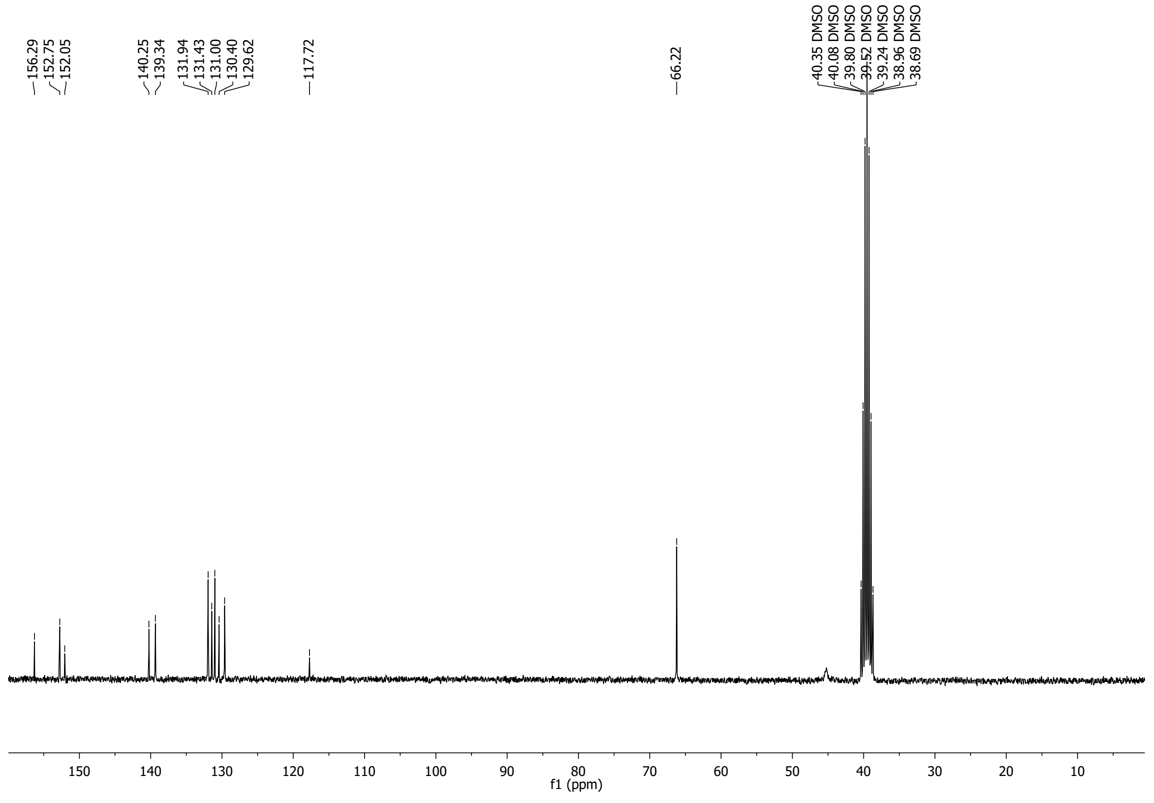

^{13}C NMR (75 MHz, DMSO-d6)

Compound 3u

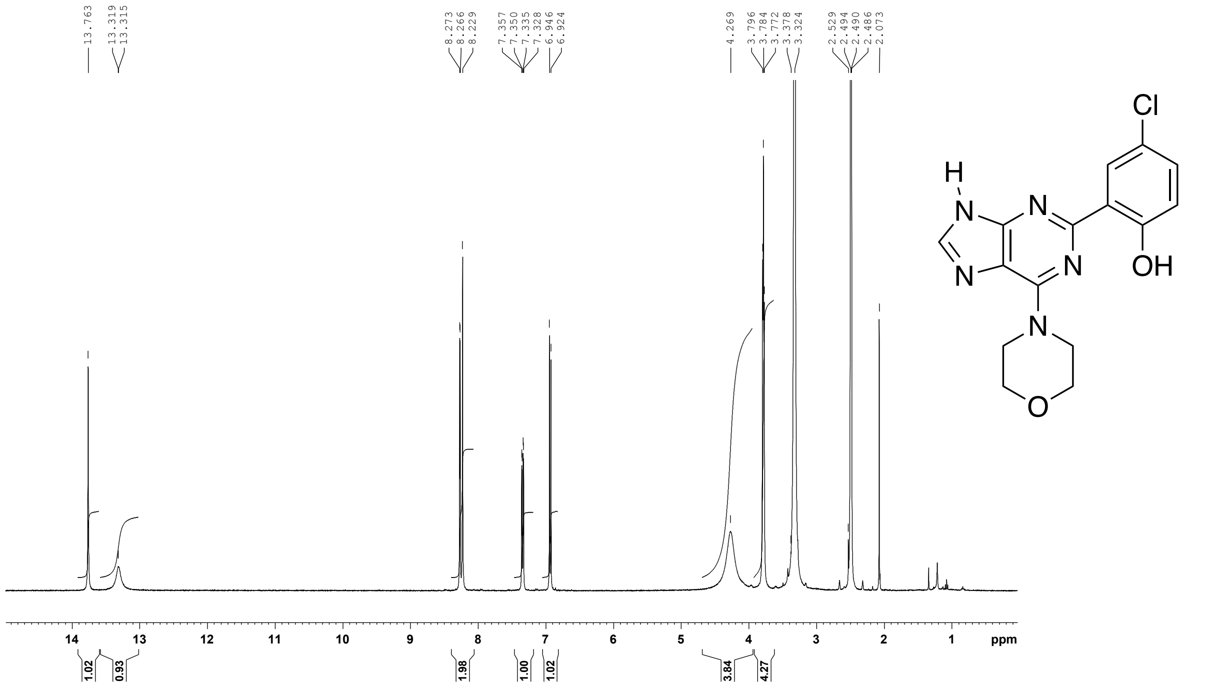

^{1}H NMR (400 MHz, DMSO-d6)

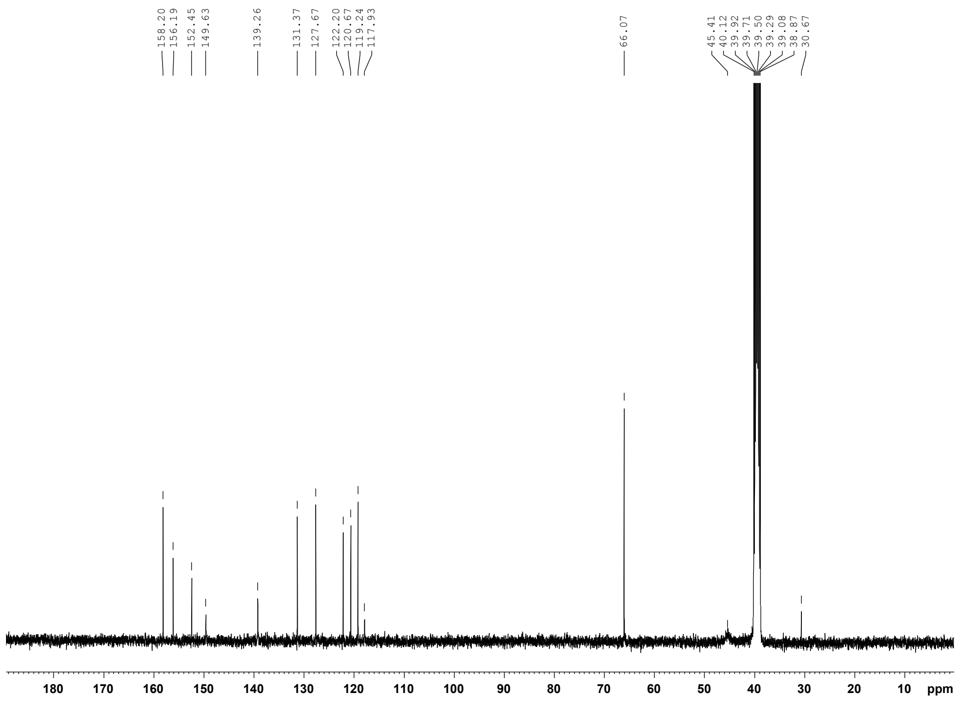

^{13}C NMR (100 MHz, DMSO-d6)

Compound 3v

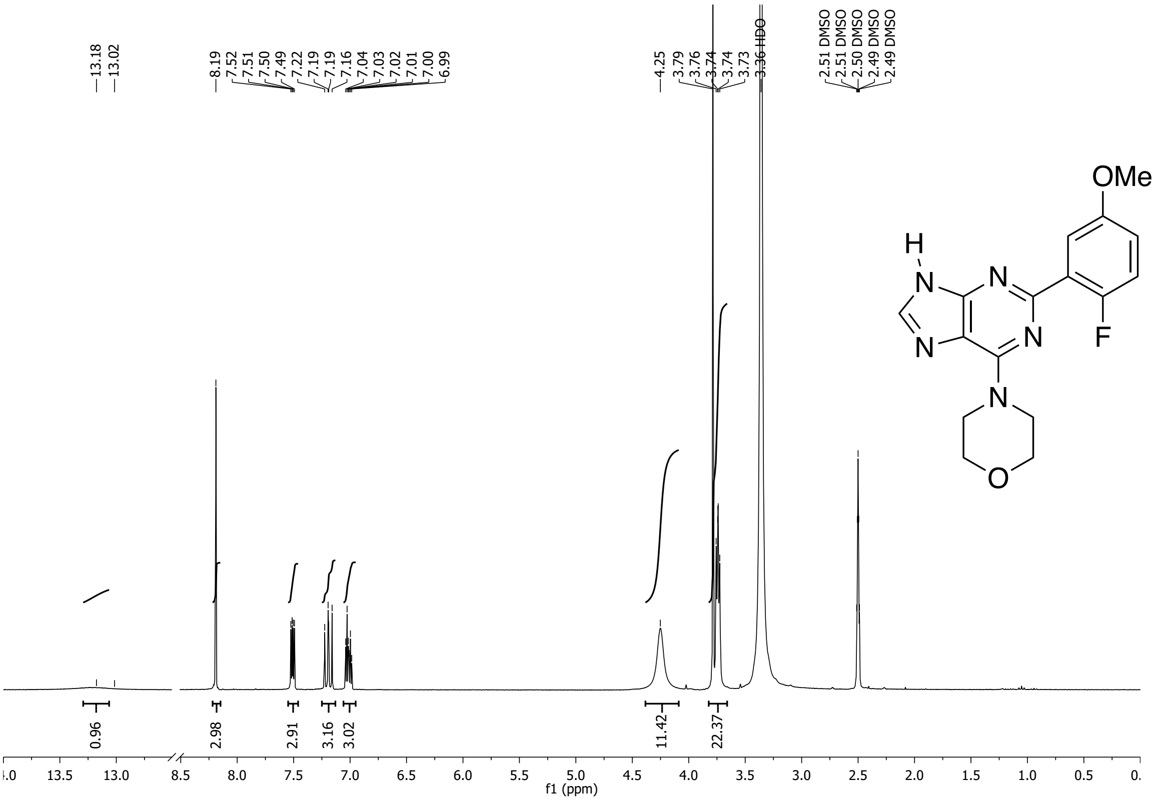

^{1}H NMR (300 MHz, DMSO-d6)

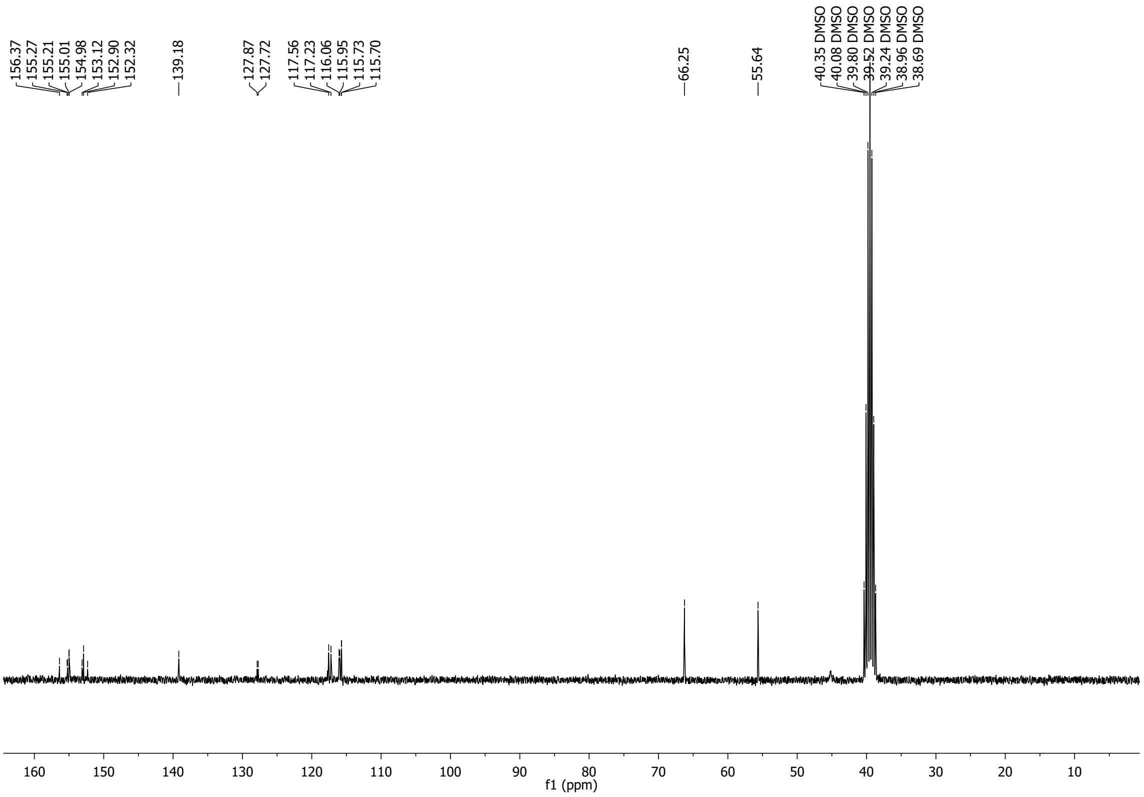

^{13}C NMR (75 MHz, DMSO-d6)
